# Predicting 30-Day Postoperative Mortality and American Society of Anesthesiologists Physical Status Using Retrieval-Augmented Large Language Models: Development and Validation Study

**DOI:** 10.2196/75052

**Published:** 2025-06-03

**Authors:** Ying-Hao Chen, Shanq-Jang Ruan, Pei-fu Chen

**Affiliations:** 1 Department of Electronic and Computer Engineering National Taiwan University of Science and Technology Taipei Taiwan; 2 Department of Anesthesiology Far Eastern Memorial Hospital New Taipei City Taiwan; 3 Department of Electrical Engineering Yuan Ze University Taoyuan Taiwan

**Keywords:** perioperative care, unstructured clinical data, machine learning, prediction model, few-shot prompting, surgical risk stratification

## Abstract

**Background:**

Accurately assessing perioperative risk is critical for informed surgical planning and patient safety. However, current prediction models often rely on structured data and overlook the nuanced clinical reasoning embedded in free-text preoperative notes. Recent advances in large language models (LLMs) have opened opportunities for harnessing unstructured clinical data, yet their application in perioperative prediction remains limited by concerns about factual accuracy. Retrieval-augmented generation (RAG) offers a promising solution—enhancing LLM performance by grounding outputs in domain-specific knowledge sources, potentially improving both predictive accuracy and clinical interpretability.

**Objective:**

This study aimed to investigate whether integrating LLMs with RAG can improve the prediction of 30-day postoperative mortality and American Society of Anesthesiologists (ASA) physical status classification using unstructured preoperative clinical notes.

**Methods:**

We conducted a retrospective cohort study using 24,491 medical records from a tertiary medical center, including preoperative anesthesia assessments, discharge summaries, and surgical information. To extract clinical insights from free-text data, we used the LLaMA 3.1-8B language model with RAG, using MedEmbed for text embedding and Miller’s Anesthesia as the primary retrieval source. We evaluated model performance under various configurations, including embedding models, chunk sizes, and few-shot prompting. Machine learning (ML) models, including random forest, support vector machines (SVM), Extreme Gradient Boosting (XGBoost), and logistic regression, were trained on structured features as baselines.

**Results:**

A total of 520 (2.1%) patients experienced in-hospital 30-day postoperative mortality. The ASA physical status distribution was as follows: class I: 535 (2.2%); class II: 15,272 (62.4%); class III: 8024 (32.8%); class IV: 606 (2.5%); and class V: 54 (0.22%). For 30-day postoperative mortality prediction, the LLaMA‑RAG model achieved an *F*_1_-score of 0.4663 (95% CI 0.4654-0.4672), versus 0.2369 (95% CI 0.2341-0.2397) without few‑shot prompting, 0.0879 (95% CI 0.0717-0.1041) without RAG, and 0.0436 (95% CI 0.0292-0.0580) without either few‑shot prompting or RAG. Among ML models, XGBoost scored 0.4459 (95% CI 0.4176-0.4742); random forest, 0.3953 (95% CI 0.3791-0.4115); logistic regression, 0.2720 (95% CI 0.2647-0.2793); and SVM, 0.2474 (95% CI 0.2275-0.2673). For ASA classification, LLaMA‑RAG achieved a micro *F*_1_-score of 0.8409 (95% CI 0.8238-0.8551) versus 0.6546 (95% CI 0.6430-0.6796) without few-shot prompting, 0.6340 (95% CI 0.6157-0.6535) without RAG, and 0.4238 (95% CI 0.3952-0.4490) without either few‑shot prompting or RAG. In comparison, XGBoost achieved 0.8273 (95% CI 0.8209-0.8498); logistic regression, 0.7940 (95% CI 0.7671-0.7950); random forest, 0.7847 (95% CI 0.7637-0.7868); and SVM, 0.7697 (95% CI 0.7637-0.7697). Notably, the model demonstrated exceptional sensitivity in identifying rare but high-risk cases, such as ASA Class 5 patients and postoperative deaths.

**Conclusions:**

The LLaMA-RAG model significantly improved the prediction of postoperative mortality and ASA classification, especially for rare high-risk cases. By grounding outputs in domain knowledge, retrieval-augmented generation enhanced both accuracy and prompt‑driven interpretability over ML and ablation models—highlighting its promise for real-world clinical decision support.

## Introduction

### Background

Nowadays, postoperative mortality remains a significant concern for health care professionals. Accurate preoperative risk prediction enables better surgical planning and anesthesia management, which can ultimately improve patient outcomes [1-5]. Existing risk assessment tools, such as the American College of Surgeons National Surgical Quality Improvement Program [6] and the American Society of Anesthesiologists physical status (ASA-PS) [7], focus primarily on structured data like patient demographics and medical history. However, these models often exclude valuable information contained in unstructured clinical text, such as surgical notes and physician reports [8].

### Prior Work

The use of machine learning (ML) in predicting postoperative mortality has shown promising results. ML models leveraging electronic health records (EHRs) have demonstrated superior predictive performance compared to traditional methods, often achieving a higher area under the receiver operating characteristic curve (AUROC) values [9-13]. Recent advancements in natural language processing, particularly the development of models like Bidirectional Encoder Representations from Transformers (BERT) [14], have improved the ability to extract meaningful insights from unstructured clinical texts [15]. However, BERT’s token length limitation constrains its ability to fully use long-form clinical notes, potentially limiting the model’s effectiveness in real-world applications [14,16].

Recent research has explored the application of large language models (LLMs) in perioperative risk prediction, specifically for predicting ASA-PS classifications. For instance, one study demonstrated that while LLMs, such as GPT-4, achieved moderate *F*_1_-scores both in hospital mortality (*F*_1_-score of 0.86) and ASA-PS predictions (*F*_1_-score of 0.50), their ability to provide interpretability and explanations for the classifications offered potential clinical utility [17]. Despite relatively lower performance in numeric scoring, the use of chain-of-thought reasoning in LLMs allowed for a better understanding of adjacent classifications, assisting clinicians in decision-making during the surgical planning phase [17].

Among LLMs, the LLaMA (LLM Meta AI) series has gained attention for its high performance, open-source availability, and adaptability across diverse natural language processing tasks [18]. LLaMA 3.1, in particular, has been shown to excel in clinical prediction tasks. For example, DRG-LLaMA, a model fine-tuned from LLaMA, demonstrated superior predictive performance for assigning diagnosis-related groups using unstructured clinical notes from the MIMIC-IV dataset. The model achieved an area under the curve of 0.986 and outperformed existing methods like ClinicalBERT, showcasing its utility in handling complex health care–related classification tasks [19].

Furthermore, a systematic review by Pressman et al [20] highlighted the broader potential of LLMs in clinical applications, including diagnostic support and risk stratification through the analysis of unstructured EHR data, underscoring the value of LLMs in perioperative care settings. These findings suggest that LLMs can complement existing risk assessment models by providing valuable insights from unstructured clinical data, despite certain limitations in numeric accuracy.

### Aim

This study aimed to demonstrate and evaluate the effectiveness of using an LLM integrated with retrieval-augmented generation (LLM-RAG) to improve clinical risk prediction, specifically for 30-day postoperative mortality and ASA-PS classification, based on preoperative unstructured clinical notes. To this end, we incorporated RAG [21], a technique designed for LLMs that dynamically retrieves and integrates relevant external information during inference. In addition to predicting postoperative mortality, we include the task of ASA-PS classification to evaluate the model’s capabilities in handling preoperative risk stratification [7,22,23]. RAG allows the model to retrieve and incorporate relevant information from external sources during inference, thereby enhancing predictive power [24].

Furthermore, this study explores both zero-shot and few-shot prediction (also known as in-context learning) strategies to investigate their respective impacts on model performance. Few-shot prompting involves providing representative tasks and solution examples in the prompt to guide the model, while zero-shot prompting provides only the query task without examples [25]. By leveraging these approaches, we aim to address the limitations of current models—particularly in handling long clinical notes, improving interpretability [24,26], and enhancing generalization across multiple tasks. Through this research, we seek to demonstrate the potential for improved prediction accuracy, model interpretability [27], and better clinical decision-making in real-world hospital settings [28]. We expect that, by improving risk prediction and enabling prompt‑driven interpretability, this LLM‑RAG approach could enhance perioperative decision support.

## Methods

### Ethical Considerations

This retrospective observational study was approved by the institutional review board of Far Eastern Memorial Hospital (112166-F), a large academic medical center. Preoperative anesthesia assessment records, surgical information, and discharge summaries were collected from the hospital’s EHR from January 1, 2016, to July 31, 2023.

### Inclusion and Exclusion Criteria

**Figure 1:**
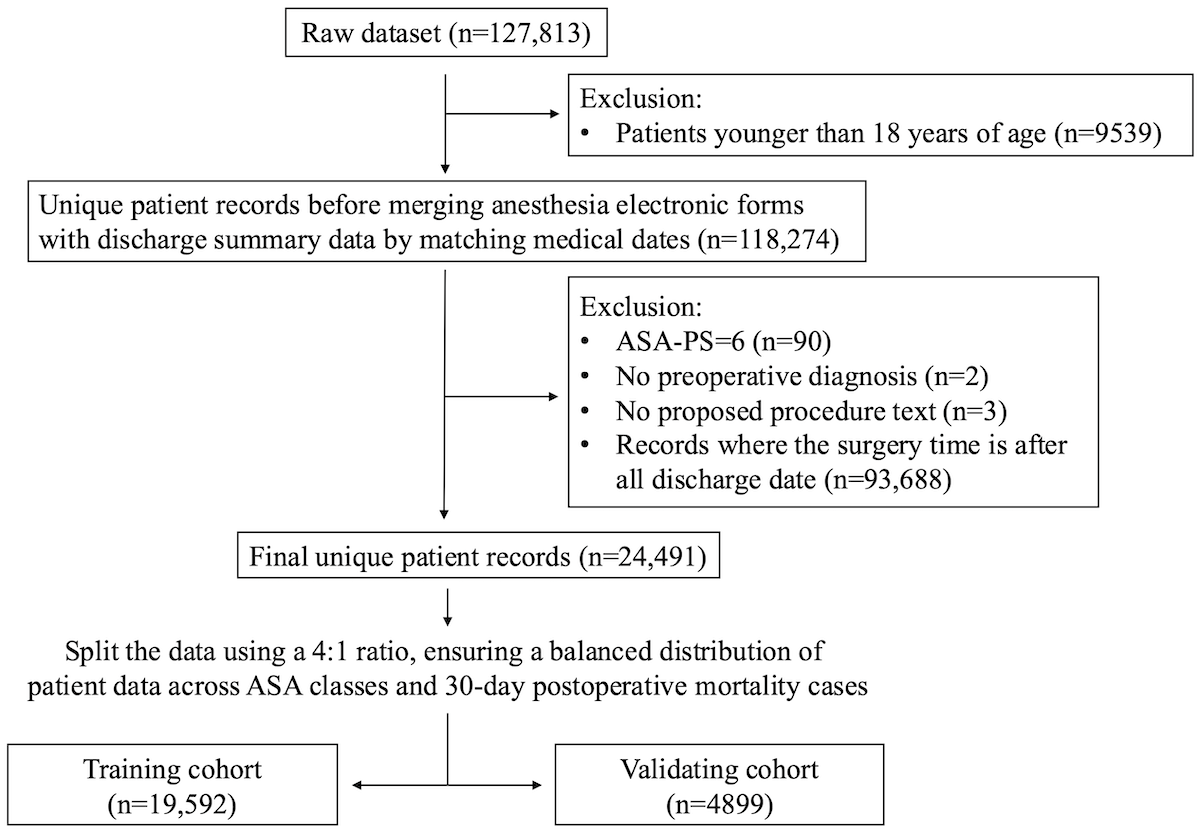
Flow diagram of data extraction and preprocessing. ASA-PS: American Society of Anesthesiologists physical status.

Patients included in this study were aged 18 years or older and had undergone at least one surgical procedure under general or neuraxial anesthesia (n=118,274). Cases were excluded if they had an ASA-PS classification of 6 (n=90), indicating patients declared brain-dead for organ donation. Additionally, records were excluded if they lacked critical information, including entry time (n=2), exit time, preoperative diagnosis, proposed procedure text (n=3), or those whose surgery date fell after all discharge dates (n=93,688). The final cohort consisted of 24,491 patients and was divided into training (n=19,592) and validation cohorts (n=4899; Figure 1).

### Outcome Definition

The primary endpoint of this study was in-hospital 30-day postoperative mortality, defined as any patient death occurring within 30 days after surgery. Mortality status was determined based on discharge records explicitly labeled as “expired” or “critical against-advice discharge” (ie, patients who left the hospital against medical advice in a critical condition, with no subsequent readmission recorded within the 30-day window). Patients discharged alive and not readmitted within 30 days were classified as nonmortality cases.

The secondary endpoint was the ASA-PS classification, recorded at the time of preoperative anesthesia evaluation. ASA-PS was categorized into five classes (labeled as 1-5), reflecting the patient’s physical condition prior to surgery [7,22,23]:

- Class 1 (ASA I): A normal, healthy patient with no systemic disease. This category includes individuals who are free from any significant health conditions and are at the lowest risk for anesthesia-related complications.
- Class 2 (ASA II): A patient with mild systemic disease that does not limit physical activity. Examples include controlled hypertension or diabetes without systemic complications, mild obesity, or smoking without chronic obstructive pulmonary disease.
- Class 3 (ASA III): A patient with severe systemic disease that limits physical activity but is not incapacitating. Conditions may include poorly controlled hypertension, diabetes with systemic complications, or a history of myocardial infarction.
- Class 4 (ASA IV): A patient with severe systemic disease that is a constant threat to life. Examples include recent myocardial infarction, unstable angina, advanced liver or kidney disease, or severe heart failure.
- Class 5 (ASA V): A moribund patient who is not expected to survive without the operation. These patients typically have life-threatening conditions such as a ruptured abdominal aneurysm or massive trauma.

### Data Preprocessing

#### Outlier Filtering

Continuous variables such as height, weight, and BMI were filtered to remove physiologically implausible values (eg, height: 120-250 cm; weight: 25-200 kg; BMI ≤100 kg/m^2^). Laboratory data, including hemoglobin, hematocrit, blood sugar, and potassium, were filtered for outliers. Vital signs, including heart rate, blood pressure, respiratory rate, body temperature, and pulse oximetry, were filtered for outliers (Multimedia Appendix 1). Outliers were identified as potential input errors and treated as missing data.

#### Structured Data Preprocessing

Structured features included patient characteristics, surgical characteristics, comorbidities, preoperative laboratory values, and preoperative vital signs (Table 1). Continuous features included age, height, weight, latest laboratory data before surgery (eg, hemoglobin, platelet count, and creatinine), and preoperative vital signs (eg, body temperature, oxygen saturation, heart rate, respiratory rate, and systolic and diastolic blood pressure). Missing values were imputed with the median value of the dataset for continuous features.

**Table 1:** The feature group included in the experiment^a^.

Feature type				Feature classes
**Structured data**				
	**Patient characteristics**			
		Continuous	Age, height, weight, BMI	
		Categorical	Sex (n=2), ASA-PS^b^ (n=5), ASA emergency (n=2), department (n=22), preoperative location (n=4), anesthesia type (n=4)	
	**Surgery characteristics**			
		Categorical	Emergency level (n=4)	
	**Comorbid conditions**			
		Categorical	Diabetes mellitus (n=2), hyperlipidemia (n=2), hypertension (n=2), cerebrovascular accident (n=2), cardiac disease (n=2), chronic obstructive pulmonary disease (n=2), asthma (n=2), hepatic disease (n=2), renal disease (n=2), bleeding disorder (n=2), major operations (n=2), smoking (n=2), drug allergy (n=2)	
	**Preoperative laboratory values**			
		Continuous	Hemoglobin, platelet, international normalized ratio, prothrombin time, activated partial thromboplastin time, creatinine, aspartate transaminase, alanine transaminase, blood sugar, serum sodium, serum potassium	
	**Preoperative vital signs**			
		Continuous	Body temperature, oxygen saturation, heart rate, respiratory rate, systolic and diastolic blood pressure	
		Categorical	Consciousness status (n=2)	
**Unstructured data**				
	Free text		Provider service, procedure, diagnosis, planned anesthesia, description, chief complaint, present illness, discharge diagnosis, discharge treatment	

^a^The parenthesized numbers indicate the number of categories within each feature class. ASA-PS classes are features for predicting mortality, but outputs for the ASA-PS classification task.

^b^ASA-PS: American Society of Anesthesiologists physical status.

Categorical features with only 2 classes, such as sex, comorbidities (eg, diabetes mellitus and hypertension), ASA emergency status, and consciousness status, were converted into binary encoding. Features with more than 2 classes, such as ASA-PS (5 classes), department (22 classes), emergency level (4 classes), preoperative location (4 classes), and anesthesia type (4 classes), were transformed into one-hot encodings. For all categorical features, missing values were imputed using the majority category from the training dataset.

#### Unstructured Data Preprocessing

Unstructured data were primarily used in the LLaMA-RAG model, leveraging LLMs, using RAG or not, and using few-shot or one-shot. These free-text clinical notes included provider service, procedure, diagnosis, emergency level, planned anesthesia, description, chief complaint, present illness, discharge diagnosis, and discharge treatment. Provider service (department), emergency level, and planned anesthesia (anesthesia type) were originally recorded in text but were also used in categorical features above after preprocessing. The integration of unstructured data allowed us to evaluate their ability to provide additional clinical insights and improve predictive accuracy beyond what structured data could achieve.

For each surgery, we extracted the latest preoperative anesthesia assessment and the discharge summary preceding the operation, along with up to four earlier summaries to capture longitudinal context. All text underwent cleaning to remove templates and headers, was converted to lowercase, stripped of punctuation, and uniformly tokenized. To meet the 4096-token limit of LLaMA 3.1‑8B, we first used a summarization prompt (Multimedia Appendix 2) to summarize each discharge note into its key clinical elements, then concatenated these summaries in chronological order. If the combined narrative exceeded the token limit, we truncated from the oldest summaries, preserving the most recent information. This pipeline distilled rich longitudinal context into a compact, structured input optimized for downstream LLM-based prediction tasks.

### Model Development

#### LLM Setup

**Figure 2:**
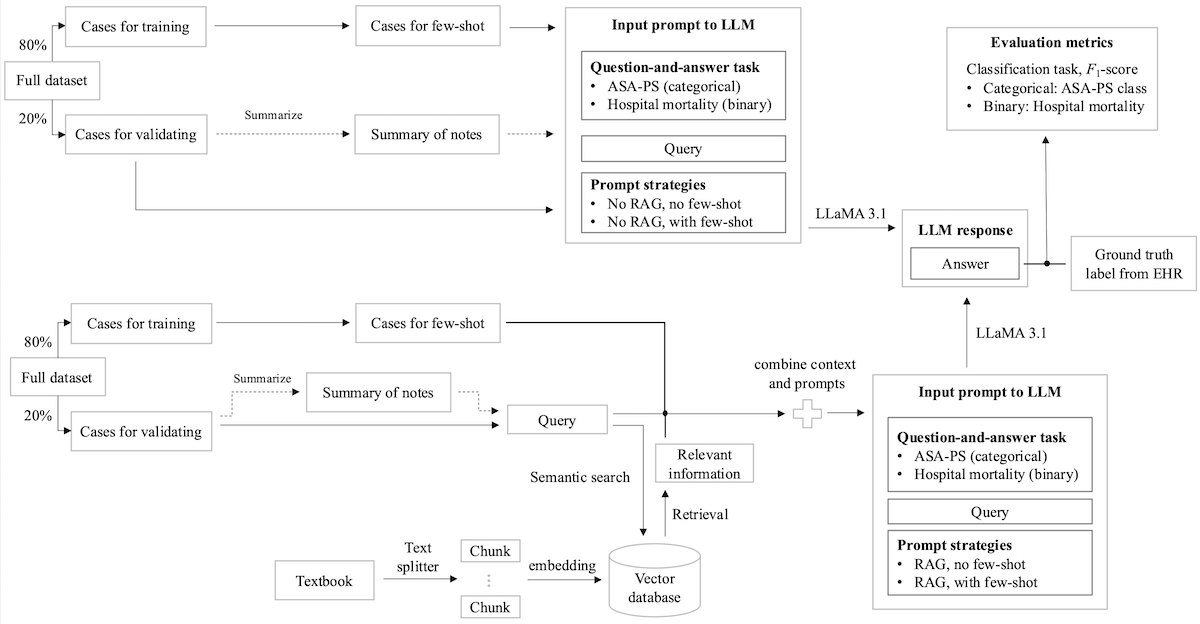
Overview of the experimental approach. The lower section illustrates the workflow with RAG, while the upper section depicts the workflow without it. In the validation workflow, cases are divided into two paths: cases with only one prior record (solid line) are directly used as prompts for the model, while cases with up to four prior records (dotted line) require summarization before being used as prompts. ASA-PS: American Society of Anesthesiologists physical status; EHR: electronic health record; LLM: large language model; RAG: retrieval-augmented generation.

Figure 2 outlines our LLaMA‑RAG experimental framework. We split the full cohort into an 80% training and a 20% validation set. The lower section illustrates the workflow with RAG and the upper section without. During validation, cases with only 1 prior record were fed directly as zero‑ or few‑shot prompts, whereas those with up to 4 prior records were first summarized using a summarization prompt and then concatenated in chronological order before prompting. For RAG configurations, each case’s query was also sent to a vector database of domain text—chunked, embedded, and retrieved via semantic search—and the top‑ranked passages were appended to the prompt.

To ensure that the selected model aligns with our needs, we conducted preliminary comparisons between LLaMA 3.1-8B and LLaMA 3.2-3B using identical prompts and hyperparameters (eg, temperature, top_p) [18]. We selected these two models due to their ability to operate efficiently on available computational resources, including the V100 graphic processing unit, 4 central processing unit cores, and 90 GB of memory. The results showed that LLaMA 3.1-8B consistently outperformed its smaller counterpart under the same conditions. Specifically, for the ASA classification task, the micro *F*_1_-score improved significantly from 0.3246 (95% CI 0.3222-0.3246) with LLaMA 3.2 to 0.4544 (95% CI 0.4531-0.4548) with LLaMA 3.1. Similarly, for the mortality prediction task, the weighted area under the precision-recall curve (AUPRC) exhibited an increase from 0.4311 (95% CI 0.4253-0.4369) with LLaMA 3.2 to 0.4959 (95% CI 0.4828-0.5090) with LLaMA 3.1 [29,30]. Other performance metrics, including precision, recall, and AUROC, also demonstrated consistent improvements and are detailed in Multimedia Appendix 3. Based on these findings, LLaMA 3.1-8B was chosen as the primary model for this study.

#### Prompting Strategies

We used four prompting strategies to evaluate the model’s performance:

- No RAG, no few-shot: Zero-shot Q&A using original clinical notes without any prior examples or retrieval.
- No RAG, with few-shot: Incorporating one or more prior examples in the prompt but without RAG.
- RAG, no few-shot: Zero-shot Q&A with RAG using external knowledge retrieved from Miller’s Anesthesia, 2-Volume Set, 9th Edition (2020) [31].
- RAG, with few-shot: Combining RAG with few-shot prompting, where external knowledge retrieved from Miller’s Anesthesia, 2-Volume Set, 9th Edition (2020) [31] is used to augment the prompt.

**Figure 3:**
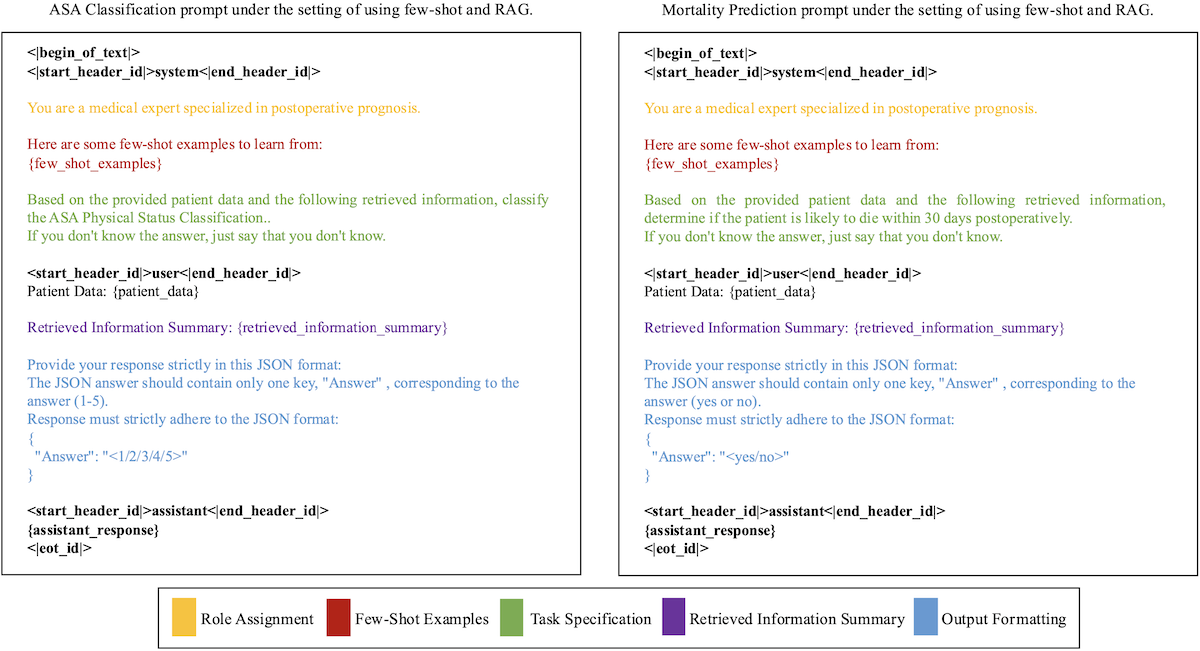
Annotated example prompt used for the mortality prediction and ASA classification tasks. When using the LLaMA model, we included the designated tags (bolded text in the figure) to ensure that the model correctly interprets and differentiates between conversational roles, thereby improving the consistency of its responses. Additionally, Role Assignment (highlighted in yellow) specifies the role that the large language model should assume when generating responses. Few-Shot Examples (highlighted in red) provide the model with example cases for learning. Task Specification explicitly defines the task that the model is required to perform. Retrieved Information Summary (highlighted in purple) represents the relevant information retrieved from the vector database based on the given patient data. Finally, Output Formatting (highlighted in blue) dictates the structure and format in which the model should present its responses, ensuring consistency with the expected output standard. For experiments conducted without few-shot learning, the Few-Shot Example section (highlighted in red) is removed from the prompt. Similarly, for experiments conducted without RAG, the Retrieved Information Summary section (highlighted in purple) is excluded. Prompts used for summary generation for Few-Shot Examples are depicted in Multimedia Appendix 2. ASA: American Society of Anesthesiologists; RAG: retrieval-augmented generation.

Prompts included structured tags for role assignment, task specification, retrieved information summaries, and output formatting. Full prompt examples are shown in Figure 3.

#### Experimental Setup

We incorporated experiments using ML models—including random forest [32], eXtreme Gradient Boosting (XGBoost) [33], logistic regression [34], and support vector machines (SVM) [35]—which used structured data as input features, in contrast to the unstructured clinical notes used by the LLM-based RAG framework. To ensure a fair and robust comparison, we systematically optimized the hyperparameters of these ML models using grid search and randomized search, implemented via the Scikit-learn library [36]. Grid Search exhaustively evaluates all possible hyperparameter combinations within a predefined space, ensuring optimal model configurations, whereas randomized search provides a computationally efficient alternative by sampling hyperparameter values from a given distribution [37]. Hyperparameter tuning was conducted using 5-fold cross-validation, optimizing for weighted AUPRC in the mortality prediction task and micro *F*_1_-score in the ASA classification task, ensuring that each model was evaluated under its best-performing configuration.

Experiments compared the LLaMA-RAG model configurations against ML baselines. Performance was benchmarked using the same training and validation cohorts, consistent preprocessing, and matched evaluation criteria. All experiments were executed on a V100 graphic processing unit with 4 central processing unit cores and 90 GB of memory.

### Model Evaluation and Statistical Analysis

This study used a retrospective cohort design with a total sample size of 24,491 surgical cases, split into a training cohort (n=19,592) and a validation cohort (n=4899). No a priori sample size or power calculation was performed; instead, the study included all eligible cases within the data collection period (January 2016-July 2023).

Model performance was assessed using the *F*_1_-score for 30-day mortality prediction and micro *F*_1_-score for ASA classification. Additional metrics, including accuracy, precision, recall, and specificity, were reported. Statistical significance of model comparisons was assessed post hoc using bootstrapping with 1000 iterations, defining significance as a 95% CI for the performance difference excluding zero (*P*<.05).

We applied Shapley Additive Explanations (SHAP) to our structured-data ML models to quantify and visualize each feature’s contribution to both 30-day postoperative mortality and ASA-PS classification, generating global importance bar plots and summary dot plots for interpretability [38].

## Results

### Outcome Distribution

In the final dataset of 24,491 unique patient records, a total of 520 (2.1%) patients experienced in-hospital 30-day postoperative mortality, including 424 (2.2%) deaths in the training (n=19,592) and 96 (2%) deaths in the validation cohort (n=4899). The ASA-PS class distributions were as follows: in the overall cohort (n=24,491), Class 1 (n=535, 2.2%), Class 2 (n=15,272, 62.4%), Class 3 (n=8024, 32.8%), Class 4 (n=606, 2.5%), and Class 5 (n=54, 0.22%); in the training cohort (n=19,592), Class 1 (n=433; 2.2%), Class 2 (n=12,207, 62%), Class 3 (n=6428, 33%), Class 4 (n=481, 2.4%), and Class 5 (n=43, 0.22%); in the validation cohort (n=4899), Class 1 (n=102, 2.1%), Class 2 (n=3065, 63%), Class 3 (n=1596, 33%), Class 4 (n=125, 2.6%), and Class 5 (n=11, 0.22%). These distributions were balanced across training and validation cohorts to support reliable model development and evaluation (Table 2 and Multimedia Appendix 4).

**Table 2:** Characteristics of the cohort^a^.

Feature				Training cohort (n=19,592)		Validating cohort (n=4899)		Overall cohort (n=24,491)
Age (years), median (IQR)				60 (47-69)		60 (47-69)		60 (47-69)
Sex (male), n (%)				11,027 (56.3)		2748 (56.1)		13,775 (56.2)
Height (cm), median (IQR)				162 (156-169)		162 (156-168)		162 (156-169)
Weight (kg), median (IQR)				65 (56.9-75)		65 (56.6-74)		65 (56.9-75)
BMI (kg/m^2^), median (IQR)				24 (22-27)		24 (22-27)		24 (22-27)
**ASA-PS^b^, n (%)**								
	1		433 (2.2)		102 (2.1)		535 (2.2)	
	2		12,207 (62)		3065 (63)		15,272 (62)	
	3		6428 (33)		1596 (33)		8024 (33)	
	4		481 (2.4)		125 (2.6)		606 (2.5)	
	5		43 (0.22)		11 (0.22)		54 (0.22)	
ASA emergency, n (%)				966 (4.9)		234 (4.8)		1200 (4.9)
30-day mortality, n (%)				424 (2.2)		96 (2)		520 (2.1)
**Emergency level of surgery, n (%)**								
	Normal		16,953 (87)		4264 (87)		21,217 (87)	
	Urgent		2046 (10)		485 (9.9)		2531 (10)	
	Emergent		543 (2.8)		143 (2.9)		686 (2.8)	
	Very emergent		48 (0.24)		7 (0.14)		55 (0.22)	
**Surgery department, n (%)**								
	Urology		4858 (24.8)		1210 (24.7)		6068 (24.8)	
	Orthopedic		3030 (15.5)		729 (14.9)		3759 (15.3)	
	General		2497 (12.7)		597 (12.2)		3094 (12.6)	
	Cardiovascular		2311 (11.8)		593 (12.1)		2904 (11.9)	
	Otolaryngology		1637 (8.4)		439 (9)		2076 (8.5)	
	Gynecology		1417 (7.2)		371 (7.6)		1788 (7.3)	
	Plastic surgery		1012 (5.2)		258 (5.3)		1270 (5.2)	
	Neurosurgery		784 (4)		190 (3.9)		974 (4)	
	Thoracic surgery		632 (3.2)		177 (3.6)		809 (3.3)	
	Colorectal surgery		390 (2)		106 (2.2)		496 (2)	
	Traumatology		320 (1.6)		63 (1.3)		383 (1.6)	
	Others		704 (3.6)		166 (3.4)		870 (3.6)	
**Comorbidity**								
		Diabetes mellitus	5016 (25.6)		1222 (24.9)		6238 (25.5)	
		Hyperlipidemia	3613 (18.4)		921 (18.8)		4534 (18.5)	
		Hypertension	9110 (46.5)		2205 (45)		11,315 (46.2)	
		Prior cerebrovascular accident	1469 (7.5)		369 (7.5)		1838 (7.5)	
		Cardiac disease	5008 (25.6)		1271 (25.9)		6279 (25.6)	
		Chronic obstructive pulmonary disease	673 (3.4)		150 (3.1)		823 (3.4)	
		Asthma	821 (4.2)		187 (3.8)		1008 (4.1)	
		Hepatic disease	2823 (14.4)		681 (13.9)		3504 (14.3)	
		Renal disease	3912 (20)		916 (18.7)		4828 (19.7)	
		Bleeding disorder	4770 (24.3)		1166 (23.8)		5936 (24.2)	
		Prior major operations	17,039 (87)		4224 (86.2)		21,263 (86.8)	
		Smoking	5297 (27)		1314 (26.8)		6611 (27)	
		Drug allergy	3683 (18.8)		877 (17.9)		4560 (18.6)	
		Consciousness	17,903 (91.4)		4507 (92)		22,410 (91.5)	
**Anesthesia type, n (%)**								
	General		17,539 (89.5)		4437 (90.6)		21,976 (89.7)	
	Neuraxial		2053 (10.5)		462 (9.4)		2515 (10.3)	
**Preoperative location, n (%)**								
		Ward	11,911 (60.8)		2998 (61.2)		14,909 (60.9)	
		Outpatient	4937 (25.2)		1228 (25.1)		6165 (25.2)	
		Emergency department	1009 (5.2)		222 (4.5)		1231 (5)	
		Intensive care unit	1678 (8.6)		435 (8.9)		2123 (8.7)	

^a^Categorical variables are represented as frequency (%) and continuous variables are represented as the median (IQR).

^b^ASA-PS: American Society of Anesthesiologists physical status.

### Preliminary Hyperparameter Tuning for the LLaMA + RAG Model

Before evaluating the performance of our proposed LLaMA + RAG model against ML approaches, we conducted a series of preliminary experiments to determine the optimal configuration for embedding models, retrieval settings, and hyperparameters. Specifically, we explored:

- Embedding models: MedEmbed [39] and PubMedBERT [40]
- Chunk sizes: 150, 200, 250, 300, and 350
- Retrieval top-k values: 6, 8, and 12
- Temperature and top-p settings: (0.001, 0.001), (0.1, 0.1), and (1, 0.5)

The goal of these experiments was to systematically identify the most effective parameter combinations for mortality prediction and ASA classification, ensuring a fair comparison between LLaMA + RAG and traditional ML models.

Our findings revealed that:

- For mortality prediction, the best-performing configuration was MedEmbed, chunk size=250, temperature=1, top_p=0.5, retrieval top_k=8.
- For ASA classification, the optimal setup was MedEmbed, chunk size=250, temperature=0.001, top_p=0.001, retrieval top_k=8.

The complete results of these parameter selection experiments, including comparisons across different settings, are provided in Multimedia Appendix 5.

With these optimal settings, we proceeded to evaluate our LLaMA + RAG model against ML algorithms, including random forest, XGBoost, SVM, and logistic regression. The following sections present a comparative analysis of mortality prediction and ASA-PS classification, demonstrating the advantages of integrating RAG with LLMs for clinical risk prediction.

### Performance Comparison

To evaluate the effectiveness of our proposed LLaMA-based method using unstructured clinical notes, we compared its performance against ML models trained on structured data. The comparison focused on mortality prediction and ASA-PS classification, using evaluation metrics including *F*_1_-score, accuracy, precision, recall, and specificity. The detailed results are presented in Table 3 for mortality prediction and Table 4 for ASA-PS classification. Additionally, a more comprehensive set of performance metrics is provided in Tables S1 and S2 in Multimedia Appendix 3 to offer deeper insights into model performance across various evaluation perspectives.

**Table 3:** Mortality prediction performances of ML^a^ models and the LLaMA-RAG^b^ model on the validation cohort.

Method		*F*_1_-score (95% CI)	Accuracy (95% CI)	Precision (95% CI)	Recall (95% CI)	Specificity (95% CI)	
**LLaMA-RAG model**		0.4663^c^ (0.4654-0.4672)	0.9580 (0.9555-0.9605)	0.3103 (0.2993-0.3213)	0.9375^c^ (0.9341-0.9409)	0.9584 (0.9562-0.9606)	
	Without few-shot, with RAG	0.2369 (0.2341-0.2397)	0.9106 (0.9087-0.9125)	0.1423 (0.1281-0.1565)	0.7083 (0.6937-0.7229)		0.9146 (0.9139-0.9153)
	With few-shot, without RAG	0.0879 (0.0717-0.1041)	0.6440 (0.6428-0.6452)	0.0463 (0.0410-0.0516)	0.8750 (0.8630-0.8870)		0.6394 (0.6387-0.6401)
	Without few-shot, without RAG	0.0436 (0.0292-0.0580)	0.5152 (0.5149-0.5155)	0.0226 (0.0114-0.0338)	0.5625 (0.5590-0.5660)		0.5143 (0.5115-0.5171)
Random forest with grid search		0.3583 (0.3391-0.3775)	0.9510 (0.9481-0.9539)	0.2410 (0.2218-0.2602)	0.6979 (0.6979-0.6979)	0.9561 (0.9540-0.9582)	
Random forest with randomized search		0.3953 (0.3791-0.4115)	0.9788^d^ (0.9766-0.9810)	0.4474^d^ (0.4360-0.4588)	0.3542 (0.3259-0.3825)	0.9913^d^ (0.9901-0.9925)	
SVM^e^ with grid search		0.2474 (0.2209-0.2739)	0.8981 (0.8966-0.8996)	0.1446 (0.1385-0.1507)	0.8542^d^ (0.8437-0.8647)	0.8991 (0.8988-0.8994)	
SVM with randomized search		0.2474 (0.2275-0.2673)	0.8981 (0.8956-0.9006)	0.1446 (0.1165-0.1727)	0.8542 (0.8478-0.8606)	0.8991 (0.8975-0.9007)	
XGBoost^f^ with grid search		0.2992 (0.2879-0.3105)	0.9245 (0.9235-0.9255)	0.1829 (0.1677-0.1981)	0.8229 (0.7965-0.8493)	0.9265 (0.9250-0.9280)	
XGBoost with randomized search		0.4459^d^ (0.4176-0.4742)	0.9822^c^ (0.9817-0.9827)	0.5738^c^ (0.5571-0.5905)	0.3646 (0.3379-0.3913)	0.9946^c^ (0.9932-0.9960)	
Logistic Regression with grid search		0.2648 (0.2492-0.2804)	0.9139 (0.9128-0.9150)	0.1590 (0.1418-0.1762)	0.7917 (0.7882-0.7952)	0.9163 (0.9158-0.9168)	
Logistic Regression with randomized search		0.2720 (0.2647-0.2793)	0.9104 (0.9076-0.9132)	0.1617 (0.1402-0.1832)	0.8542^d^ (0.8538-0.8546)	0.9115 (0.9114-0.9116)	

^a^ML: machine learning.

^b^RAG: retrieval-augmented generation.

^c^Indicates best performance in each column.

^d^Indicates second-best performance.

^e^SVM: support vector machine.

^f^XGBoost: extreme gradient boosting.

**Table 4:** ASA^a^ classification performances of ML^b^ models and LLaMA-RAG^c^ model on the validation cohort.

Method			Micro *F*_1_-score (95% CI)		Accuracy (95% CI)		Precision (95% CI)		Recall (95% CI)		Specificity (95% CI)
**LLaMA-RAG model**			0.8409^d^ (0.8238-0.8551)		0.7409 (0.7381-0.7435)		0.8125 (0.7832-0.8418)		0.7409 (0.7370-0.7448)		0.8836 (0.8834-0.8838)
	Without few-shot, with RAG	0.6546 (0.6430-0.6796)		0.6546 (0.6534-0.6573)		0.8113 (0.7986-0.8240)		0.6546 (0.6545-0.6547)		0.9065^d^ (0.9039-0.9091)	
	With few-shot, without RAG	0.6340 (0.6157-0.6535)		0.6340 (0.6328-0.6350)		0.7501 (0.7380-0.7622)		0.6340 (0.6087-0.6593)		0.8076 (0.8075-0.8077)	
	Without few-shot, without RAG	0.4238 (0.3952-0.4490)		0.4239 (0.4225-0.4251)		0.6834 (0.6659-0.7009)		0.4239 (0.3975-0.4503)		0.7199 (0.7185-0.7213)	
SVM^e^ with grid search			0.7697 (0.7637-0.7697)		0.7696 (0.7675-0.7710)		0.8054 (0.7930-0.8178)		0.7697 (0.7590-0.7804)		0.8450 (0.8449-0.8451)
SVM with randomized search			0.7250 (0.7068-0.7364)		0.7250 (0.7238-0.7268)		0.8015 (0.7948-0.8082)		0.7250 (0.6994-0.7506)		0.8569^f^ (0.8566-0.8572)
XGBoost^g^ with grid search			0.8273^f^ (0.8209-0.8498)		0.8273^d^ (0.8253-0.8286)		0.8178^d^ (0.8136-0.8220)		0.8273^d^ (0.8065-0.8481)		0.8050 (0.8044-0.8056)
XGBoost with randomized search			0.8247 (0.8127-0.8401)		0.8247^f^ (0.8221-0.8272)		0.8173^f^ (0.7999-0.8347)		0.8247^f^ (0.8241-0.8253)		0.8011 (0.8011-0.8011)
Logistic regression with grid search			0.7940 (0.7671-0.7950)		0.7940 (0.7930-0.7953)		0.8063 (0.7803-0.8323)		0.7940 (0.7899-0.7981)		0.8314 (0.8291-0.8337)
Logistic regression with randomized search			0.7867 (0.7700-0.8062)		0.7867 (0.7848-0.7874)		0.8074 (0.7960-0.8188)		0.7867 (0.7755-0.7979)		0.8369 (0.8360-0.8378)

^a^ASA: American Society of Anesthesiologists.

^b^ML: machine learning.

^c^RAG: retrieval-augmented generation.

^d^Indicates best performance in each column.

^e^SVM: support vector machine.

^f^Indicates second-best performance.

^g^XGBoost: extreme gradient boosting.

### Mortality Prediction Task

Our LLaMA-RAG model achieved the highest *F*_1_-score of 0.4663 (95% CI 0.4654-0.4672) and recall of 0.9375 (95% CI 0.9341-0.9409), indicating its strong capability in correctly identifying mortality cases (Table 3). Among ML models, XGBoost with randomized search attained the second-highest *F*_1_-score of 0.0.4459 (95% CI 0.4176-0.4742), while SVM with randomized search obtained the second-highest recall of 0.8542 (95% CI 0.8478-0.8606). In ablation experiments, the *F*_1_-score dropped to 0.2369 (95% CI 0.2341-0.2397) without few‑shot prompting, 0.0879 (95% CI 0.0717-0.1041) without RAG, and 0.0436 (95% CI 0.0292-0.0580) without either few-shot prompting or RAG.

Attention heatmaps reveal that, for mortality prediction, the LLaMA-RAG model consistently concentrates its attention on the “present illness” section, often supplemented by the “discharge diagnosis” field, underscoring acute clinical trajectory as the primary driver of mortality risk estimation (Multimedia Appendices 6 and 7). SHAP analysis of the XGBoost revealed that hemoglobin was the single most influential predictor—lower values markedly increasing predicted risk—followed by intensive care unit, tachycardia, and serum sodium, all of which exhibited the highest mean absolute SHAP values among the top 20 features (Multimedia Appendix 8).

### ASA Classification Task

For ASA classification, our LLaMA-RAG model outperformed ML models, achieving the highest micro *F*_1_-score of 0.8409 (95% CI 0.8238-0.8551) and specificity of 0.8836 (95% CI 0.8834-0.8838), detailed in Table 4. Among traditional methods, XGBoost with grid search achieved the second-highest micro *F*_1_-score of 0.8273 (95% CI 0.8209-0.8498), whereas SVM with randomized search obtained the second-highest specificity of 0.8569 (95% CI 0.8566-0.8572). In ablation experiments, the micro *F*_1_-score dropped to 0.6546 (95% CI 0.6430-0.6796) without few‑shot prompting, 0.6340 (95% CI 0.6157-0.6535) without RAG, and 0.4238 (95% CI 0.3952-0.4490) without either few-shot prompting or RAG.

Attention heatmaps for ASA-PS classification show that the LLaMA-RAG model consistently focuses its attention on the “present illness” section, often with additional peaks on “discharge diagnosis” and “discharge treatment,” indicating that the model leverages the patient’s most recent clinical trajectory and outcomes when assigning ASA-PS (Multimedia Appendices 6 and 7). SHAP analysis of the XGBoost revealed that age was the single most influential predictor, lower values markedly decreased predicted class, followed by elective surgery, cardiac disease, and cardiovascular surgery, all of which exhibited the highest mean absolute SHAP values among the top 20 features (Multimedia Appendix 8).

## Discussion

### Principal Results

This study demonstrates the effectiveness of integrating an LLM with RAG for clinical risk prediction, achieving superior performance in both mortality prediction and ASA classification.

In the mortality prediction task, our LLaMA-RAG model obtained the highest AUPRC of 0.6536 (95% CI 0.6479-0.6593), AUROC of 0.9570 (95% CI 0.9543-0.9597), and *F*_1_-score of 0.4663 (95% CI 0.4654-0.4672), significantly outperforming ML models, including random forest, SVM, XGBoost, and logistic regression in AUPRC (Multimedia Appendix 9). While our AUROC score was significantly higher than that of SVM and logistic regression, it remained comparable to that of random forest and XGBoost (Table 3). For ASA multiclassification, our method achieved the highest micro *F*_1_-score of 0.8409 (95% CI 0.8238-0.8551), outperforming all other ML models (Table 4). In ablation experiments for both tasks, removal of either RAG or few‑shot prompting led to substantial declines in the *F*_1_-score (Tables 3 and 4), underscoring the critical contributions of both components to the model’s discriminative performance.

However, while our approach demonstrated advantages in AUPRC, AUROC, and recall, it did not surpass all ML baselines in terms of accuracy and precision. A closer examination of confusion matrices (Tables S1 and S2 in Multimedia Appendix 3) provides insight into this result: our model exhibited superior sensitivity in identifying rare cases, such as ASA Class 5 patients and postoperative mortality. Even when misclassifications occurred, they were predominantly within adjacent ASA classes, which may reflect the inherent subjectivity in ASA grading (Multimedia Appendix 10). This suggests that our method has the capability to perform clinical risk stratification.

### Comparison With Prior Works

Our method also outperformed the approach of Chung et al [17], which used ChatGPT-4 Turbo for ASA classification, achieving a micro *F*_1_-score of 0.8409 (95% CI 0.8238-0.8551), compared to their best result of 0.54 (95% CI 0.49-0.60). This difference highlights the limitations of a general-domain LLM without structured retrieval augmentation. While GPT-4 Turbo benefits from extensive pretraining on diverse text sources, its lack of domain-specific retrieval may restrict its ability to accurately capture complex clinical relationships. By contrast, our retrieval-enhanced model systematically integrates domain knowledge, leading to a more interpretable and clinically reliable risk stratification process [41,42].

When compared to prior deep learning–based approaches, our method significantly outperforms the BERT–deep neural network (DNN) model of Chen et al [43] in mortality prediction. Specifically, our LLM with RAG integration achieved a substantially higher macro *F*_1_-score (0.7222, 95% CI 0.6998-0.7446) compared to their model (0.307, 95% CI 0.269-0.342), indicating a superior balance between precision and recall across both mortality and survival classes. Unlike the BERT-DNN framework, which relies solely on parametric memory for text extraction, our approach dynamically retrieves specialized anesthesia knowledge from textbooks [31]. This retrieval mechanism not only enhances contextual understanding but also mitigates the risk of hallucinated responses common in LLMs [41,42]. By leveraging domain-specific knowledge, our method achieves more reliable and clinically relevant predictions.

In addition to performance comparisons, it is important to note that our study leveraged a substantially larger and more diverse dataset (n=24,491) compared to Chung et al [17], whose task-specific datasets were limited to 1000 cases each. This broader dataset allowed us to capture a wider range of surgical procedures, patient backgrounds, and rare outcomes, enhancing the generalizability of our model. Furthermore, unlike Chung et al [17], who focused primarily on few-shot prompting strategies without retrieval augmentation, our integration of RAG provided domain-specific knowledge, significantly improving predictive accuracy and interpretability. Although Chen et al [43] trained a BERT-DNN model on 121,313 cases, their approach relied on structured inputs and brief diagnostic and procedural snippets (less than 50 tokens) without RAG. By contrast, our framework leveraged both structured features and rich, LLM-summarized discharge narratives to evaluate LLaMA-RAG for 30-day mortality and ASA-PS prediction.

### Key Factors Influencing Model Performance

Our findings highlight the critical role of RAG in improving LLM-driven clinical predictions. Traditional LLMs rely solely on their pretraining corpus, which limits their ability to provide up-to-date and domain-specific medical insights. By dynamically retrieving relevant knowledge from trusted medical references, RAG ensures that predictions remain factually grounded and clinically relevant [41,42]. This advantage was particularly evident in mortality prediction, where the incorporation of RAG helped mitigate class imbalance issues, leading to improved AUPRC and predictive performance (Table 3).

To further investigate the effectiveness of RAG-enhanced LLMs, we systematically evaluated 3 key factors influencing model performance: embedding model selection, chunk size configuration, and few-shot prompting strategies (Table S3 in Multimedia Appendix 3 and Multimedia Appendix 11).

### Impact of Embedding Model Selection

We first compared MedEmbed and PubMedBERT under identical hyperparameter settings to assess their impact on model performance. Our results demonstrated that MedEmbed consistently outperformed PubMedBERT, achieving the highest AUPRC of 0.4733 for mortality prediction and the highest micro *F*_1_-score of 0.6307 for ASA classification (Figures S1 and S2 in Multimedia Appendix 5). These findings suggest that embedding models optimized for medical text representations can significantly enhance LLM-based clinical predictions.

### Effect of Chunk Size on Model Performance

Next, we examined the influence of chunk size selection in retrieval-based augmentation. Our experiments revealed that a chunk size of 250 yielded the best performance across both tasks. For mortality prediction, the highest AUPRC (0.4733) was obtained at retrieval top_k=12, whereas for ASA classification, the best micro *F*_1_-score (0.6307) was achieved at retrieval top_k=6 (Figures S3 and S4 in Multimedia Appendix 5). These results emphasize that optimizing the chunking strategy when embedding clinical texts plays a crucial role in maximizing retrieval effectiveness and overall predictive accuracy.

### Influence of Few-Shot Learning on Performance

We further assessed the impact of few-shot prompting, which provided additional performance gains. For mortality prediction, incorporating 9-shot examples resulted in the highest AUPRC of 0.6536, compared to 0.6176 with 5-shot prompting. In ASA classification, few-shot learning significantly improved model performance, yielding a micro *F*_1_-score of 0.8409, a substantial increase over the 0.5938 obtained without few-shot prompting (Figures S5 and S6 in Multimedia Appendix 5). These findings underscore the importance of carefully curating Few-Shot Examples to enhance the generalizability of LLM-based clinical models.

### Implications for Clinical Artificial Intelligence

Collectively, our findings highlight the benefits of integrating RAG with LLMs, selecting an effective embedding model, optimizing chunk size, and leveraging few-shot prompting to improve clinical prediction performance. The ability to dynamically retrieve domain-specific knowledge makes RAG-enhanced LLMs particularly well-suited for real-world medical decision-making, where contextual interpretation of patient characteristics and clinical guidelines is essential (Multimedia Appendix 12). This capability is particularly advantageous in medical domains where well-established guidelines and textbooks serve as primary decision-making references. Unlike conventional LLMs that rely solely on pretraining data, RAG dynamically retrieves up-to-date clinical knowledge, ensuring that predictions and recommendations remain aligned with current best practices. By mitigating the risks of outdated information and hallucinated responses, RAG enhances the reliability and interpretability of artificial intelligence (AI)–driven decision support.

Additionally, our confusion matrices suggest that while our method may not always outperform traditional ML models in precision and accuracy, it excels at identifying rare but clinically significant cases, such as ASA Class 5 patients and postoperative mortality events (Multimedia Appendix 13). For ASA class 5, the full LLaMA-RAG model achieved a true positive rate of 54.5%, compared with 54.5% without RAG, 27.3% without few-shot prompting, and 9.1% without either component; traditional ML models identified no class V cases (0% true positives). For 30-day mortality, LLaMA-RAG reached a true positive rate of 94.8%, versus 87.5% without RAG, 79.2% without few-shot prompting, and 56.2% without either, while ML baselines ranged from 35.4% to 85.4%. These results demonstrate that both RAG and few-shot prompting incrementally enhance the LLaMA-RAG model’s ability to detect rare but clinically significant outcomes. This highlights the trade-off between precision and recall in clinical decision support systems, emphasizing the need to balance risk prediction sensitivity and specificity in practice.

Moreover, these results underscore the transformative potential of LLM integrated with RAG architectures in clinical informatics. By improving both discrimination and interpretability, RAG-enhanced LLMs provide a more reliable and adaptable framework for clinical AI applications, bridging the gap between machine intelligence and human expertise in medical practice.

### Limitations

This study used a fixed-length chunking strategy for RAG, which may misalign with the semantic structure of a clinical text. Semantic chunking, leveraging meaning-based segmentation, could enhance retrieval relevance [44,45]. Adaptive retrieval, incorporating hybrid query-based methods, may further optimize context selection [45]. Additionally, Adaptive RAG, which adjusts retrieval depth based on query complexity, remains unexplored in our setting but has shown promise in dynamically optimizing information retrieval [46]. Future studies should systematically evaluate these techniques to improve retrieval precision, computational efficiency, and applicability in clinical AI systems.

### Conclusions

This study demonstrates the effectiveness of integrating LLMs with RAG for clinical risk prediction. Our LLaMA-RAG model demonstrated superior performance compared to both ML models and previously published deep learning approaches in mortality prediction and ASA classification [17,43], especially for rare high‑risk patients, by grounding its analysis in reliable medical knowledge to deliver more accurate, interpretable, and clinically relevant predictions. Through systematic evaluations of embedding model selection, chunk size optimization, and few-shot prompting, we identified key factors influencing predictive accuracy. RAG-enhanced LLMs provide a promising pathway toward more interpretable, context-aware, and accurate clinical decision support systems.

## Appendix

Multimedia Appendix 1 Continuous feature limits to define outliers.

Multimedia Appendix 2 Example prompt template used for summarizing patient records with multiple prior discharge notes, illustrating role assignment, task specification, and structured output formatting for use in few-shot learning.

Multimedia Appendix 3 Comprehensive performance metrics for all models across mortality prediction and ASA classification tasks, including accuracy, precision, recall, F1-scores, AUROC, AUPRC, and confusion matrices (Tables S1 and S2), as well as comparative results evaluating the effects of embedding models, chunk sizes, and few-shot prompting on model performance (Table S3). ASA: American Society of Anesthesiologists; AUROC: area under the receiver operating characteristic curve; AURPC: area under the precision-recall curve.

Multimedia Appendix 4 Summary of laboratory and vital sign values.

Multimedia Appendix 5 Comparative analysis of embedding models, chunk sizes, and few-shot prompting strategies in retrieval-augmented clinical risk prediction tasks. Results include their impact on AUPRC, F1 scores, and recall for both mortality prediction and ASA classification. ASA: American Society of Anesthesiologists; AURPC: area under the precision-recall curve.

Multimedia Appendix 6 Normalized token-level attention weights visualized for example cases in 30-day postoperative mortality prediction and ASA-PS classification tasks. ASA-PS: American Society of Anesthesiologists physical status.

Multimedia Appendix 7 Representative preoperative assessment and discharge summary excerpts used to generate the attention heatmaps in Multimedia Appendix 11.

Multimedia Appendix 8 Global explainability plots for XGBoost models, including (A) mean absolute SHAP value bar chart and (B) SHAP summary dot plot, for both mortality prediction and ASA-PS classification. ASA-PS: American Society of Anesthesiologists physical status; SHAP: Shapley Additive Explanations; XGBoost: Extreme Gradient Boosting.

Multimedia Appendix 9 Statistical significance testing of model performance differences for mortality prediction (AUPRC) and ASA classification (Micro F1 Score), based on 1,000 test set resamples and pairwise comparisons with the proposed method. ASA: American Society of Anesthesiologists; AUPRC: area under the precision-recall curve.

Multimedia Appendix 10 Misclassification analysis for ASA Class 5 patients, comparing adjacent and distant errors across models. Results highlight the rate and severity of ASA classification errors, particularly in high-risk cases. ASA: American Society of Anesthesiologists.

Multimedia Appendix 11 Confusion matrices for mortality prediction and ASA classification across various model configurations, including comparisons of machine learning baselines, LLaMA variants, RAG integration, and few-shot prompting strategies. ASA: American Society of Anesthesiologists; RAG: Retrieval-Augmented Generation.

Multimedia Appendix 12 Sample LLaMA-RAG model outputs for ASA classification and mortality prediction, illustrating how the model generates clinically grounded justifications based on patient history. ASA: American Society of Anesthesiologists; RAG: Retrieval-Augmented Generation.

Multimedia Appendix 13 True positive rates for ASA Class 5 and postoperative mortality across baseline machine learning models, illustrating detection performance for rare but clinically critical cases. ASA: American Society of Anesthesiologists.

## Abbreviations

AI: artificial intelligence
ASA-PS: American Society of Anesthesiologists physical status
AUPRC: area under the precision-recall curve
AUROC: area under the receiver operating characteristic curve
BERT: Bidirectional Encoder Representations from Transformers
DNN: deep neural network
EHR: electronic health record
LLM: large language model
ML: machine learning
RAG: Retrieval-Augmented Generation
SHAP: Shapley Additive Explanations
SVM: support vector machine
XGBoost: Extreme Gradient Boosting

## Data Availability

The datasets generated or analyzed during this study are not publicly available due to Institutional Review Board restrictions that prohibit open sharing of patient-level data to protect participant privacy but are available from the corresponding author upon reasonable request.

## References

[1] Weiser TG, Regenbogen SE, Thompson KD, Haynes AB, Lipsitz SR, Berry WR, Gawande AA (2008). An estimation of the global volume of surgery: a modelling strategy based on available data. Lancet.

[2] Pearse RM, Moreno RP, Bauer P, Pelosi P, Metnitz P, Spies C, Vallet B, Vincent J, Hoeft A, Rhodes A (2012). Mortality after surgery in Europe: a 7 day cohort study. Lancet.

[3] Bronsert MR, Lambert-Kerzner A, Henderson WG, Hammermeister KE, Atuanya C, Aasen DM, Singh AB, Meguid RA (2020). The value of the "Surgical Risk Preoperative Assessment System" (SURPAS) in preoperative consultation for elective surgery: a pilot study. Patient Saf Surg.

[4] Hughes MJ, McNally S, Wigmore SJ, Deans D, Skipworth RJ (2021). Preoperative risk stratification: identifying modifiable risks for surgical patients. Perioperative Medicine.

[5] Firde M, Yetneberk T (2024). Preoperative investigation practices for elective surgical patients: clinical audit. BMC Anesthesiol.

[6] Khuri S (2005). The NSQIP: a new frontier in surgery. Surgery.

[7] Mayhew D, Mendonca V, Murthy BVS (2019). A review of ASA physical status—historical perspectives and modern developments. Anaesthesia.

[8] Shickel B, Tighe PJ, Bihorac A, Rashidi P (2018). Deep EHR: A survey of recent advances in deep learning techniques for electronic health record (EHR) analysis. IEEE J Biomed Health Inform.

[9] Corey KM, Kashyap S, Lorenzi E, Lagoo-Deenadayalan SA, Heller K, Whalen K, Balu S, Heflin MT, McDonald SR, Swaminathan M, Sendak M (2018). Development and validation of machine learning models to identify high-risk surgical patients using automatically curated electronic health record data (Pythia): a retrospective, single-site study. PLoS Med.

[10] Rajkomar A, Oren E, Chen K, Dai AM, Hajaj N, Hardt M, Liu PJ, Liu X, Marcus J, Sun M, Sundberg P, Yee H, Zhang K, Zhang Y, Flores G, Duggan GE, Irvine J, Le Q, Litsch K, Mossin A, Tansuwan J, Wang D, Wexler J, Wilson J, Ludwig D, Volchenboum SL, Chou K, Pearson M, Madabushi S, Shah NH, Butte AJ, Howell MD, Cui C, Corrado GS, Dean J (2018). Scalable and accurate deep learning with electronic health records. NPJ Digital Med.

[11] Soguero-Ruiz C, Hindberg K, Mora-Jiménez I, Rojo-Álvarez JL, Skrøvseth SO, Godtliebsen F, Mortensen K, Revhaug A, Lindsetmo R, Augestad KM, Jenssen R (2016). Predicting colorectal surgical complications using heterogeneous clinical data and kernel methods. J Biomed Inform.

[12] Weller GB, Lovely J, Larson DW, Earnshaw BA, Huebner M (2018). Leveraging electronic health records for predictive modeling of post-surgical complications. Stat Methods Med Res.

[13] Ren Y, Loftus TJ, Datta S, Ruppert MM, Guan Z, Miao S, Shickel B, Feng Z, Giordano C, Upchurch GR, Rashidi P, Ozrazgat-Baslanti T, Bihorac A (2022). Performance of a machine learning algorithm using electronic health record data to predict postoperative complications and report on a mobile platform. JAMA Netw Open.

[14] Devlin J, Chang MW, Lee K, Toutanova K (2019). BERT: pre-training of deep bidirectional transformers for language understanding.

[15] Raza S, Schwartz B (2023). Entity and relation extraction from clinical case reports of COVID-19: a natural language processing approach. BMC Med Inform Decis Mak.

[16] Su P, Vijay-Shanker K (2022). Investigation of improving the pre-training and fine-tuning of BERT model for biomedical relation extraction. BMC Bioinformatics.

[17] Chung P, Fong CT, Walters AM, Aghaeepour N, Yetisgen M, O'Reilly-Shah VN (2024). Large language model capabilities in perioperative risk prediction and prognostication. JAMA Surg.

[18] Grattafiori A, Dubey A, Jauhri A, Pandey A, Kadian A, Al-Dahle A, Letman A, Mathur A, Schelten A (2024). The Llama 3 herd of models. ArXiv. Preprint posted online on July 31, 2024.

[19] Wang H, Gao C, Dantona C, Hull B, Sun J (2024). DRG-LLaMA : tuning LLaMA model to predict diagnosis-related group for hospitalized patients. NPJ Digital Med.

[20] Pressman SM, Borna S, Gomez-Cabello CA, Haider SA, Haider CR, Forte AJ (2024). Clinical and surgical applications of large language models: a systematic review. J Clin Med.

[21] Lewis P, Perez E, Piktus A, Petroni F, Karpukhin V, Goyal N (2020). Retrieval-augmented generation for knowledge-intensive NLP tasks. Adv Neural Inf Process Syst.

[22] Saklad M (1941). Grading of patients for surgical procedures. Anesthesiology.

[23] Horvath B, Kloesel B, Todd M, Cole D, Prielipp R (2021). The evolution, current value, and future of the American Society of Anesthesiologists physical status classification system. Anesthesiology.

[24] Hao B, Zhu H, Paschalidis I (2020). Enhancing clinical BERT embedding using a biomedical knowledge base.

[25] Alayrac JB, Donahue J, Luc P, Miech A, Barr I, Hasson Y, Lenc K (2022). Flamingo: a visual language model for few-shot learning. ArXiv. Preprint posted online on April 29, 2022.

[26] Lee J, Yoon W, Kim S, Kim D, Kim S, So CH, Kang J (2020). BioBERT: a pre-trained biomedical language representation model for biomedical text mining. Bioinformatics.

[27] Zhang Y, Chen Q, Yang Z, Lin H, Lu Z (2019). BioWordVec, improving biomedical word embeddings with subword information and MeSH. Sci Data.

[28] Wang Y, Zhang L, Zhang Y, Xia Y, Yang Y, Guo J (2021). Incorporating domain knowledge into BERTvaluating the impact of knowledge sources on medical relation extraction. J Biomed Inform.

[29] Saito T, Rehmsmeier M (2015). The precision-recall plot is more informative than the ROC plot when evaluating binary classifiers on imbalanced datasets. PLoS One.

[30] Ozenne B, Subtil F, Maucort-Boulch D (2015). The precision-recall curve overcame the optimism of the receiver operating characteristic curve in rare diseases. J Clin Epidemiol.

[31] Miller RD, Cohen NH, Eriksson LI, Fleisher LA, Leslie K, Wiener-Kronish JP (2020). Miller's Anesthesia, 2-Volume Set.

[32] Breiman L (2001). Random Forests. Mach Learn.

[33] Chen T, Guestrin C (2016). XGBoost: a scalable tree boosting system.

[34] Cox D (1958). The regression analysis of binary sequences. J R Stat Soc Series B Stat Methodol.

[35] Cortes C, Vapnik V (1995). Support-vector networks. Mach Learn.

[36] Pedregosa F, Varoquaux G, Gramfort A, Michel V, Thirion B, Grisel O, Blondel M, Prettenhofer P, Weiss R, Dubourg V, Vanderplas V, Passos A, Cournapeau D, Brucher M, Perrot M (2011). Scikit-learn: machine learning in python. J Mach Learn Res.

[37] Bergstra J, Bengio Y (2012). Random search for hyper-parameter optimization. J Mach Learn Res.

[38] Lundberg SM, Erion G, Chen H, DeGrave A, Prutkin JM, Nair B, Katz R, Himmelfarb J, Bansal N, Lee S (2020). From local explanations to global understanding with explainable AI for trees. Nat Mach Intell.

[39] Abhinand B (2024). MedEmbed: medical-focused embedding models. Github.

[40] Gu Y, Tinn R, Cheng H, Lucas M, Usuyama N, Liu X, Naumann T, Gao J, Poon H (2021). Domain-specific language model pretraining for biomedical natural language processing. ACM Trans Comput Healthcare.

[41] Zakka C, Shad R, Chaurasia A, Dalal AR, Kim JL, Moor M, Fong R, Phillips C, Alexander K, Ashley E, Boyd J, Boyd K, Hirsch K, Langlotz C, Lee R, Melia J, Nelson J, Sallam K, Tullis S, Vogelsong MA, Cunningham JP, Hiesinger W (2024). Almanac—retrieval-augmented language models for clinical medicine. NEJM AI.

[42] Unlu O, Shin J, Mailly CJ, Oates MF, Tucci MR, Varugheese M, Wagholikar K, Wang F, Scirica BM, Blood AJ, Aronson SJ (2024). Retrieval-augmented generation–enabled GPT-4 for clinical trial screening. NEJM AI.

[43] Chen PF, Chen L, Lin YK, Li GH, Lai F, Lu CW, Yang CY, Chen KC, Lin TY (2022). Predicting postoperative mortality with deep neural networks and natural language processing: model development and validation. JMIR Med Inform.

[44] Mahboub A, Za'ter M, Al-Rfooh B, Estaitia Y, Jaljuli A, Hakouz A (2024). Evaluation of semantic search and its role in retrieved-augmented-generation (RAG) for arabic language. ArXiv. Preprint posted online on March 27, 2024.

[45] Sawarkar K, Mangal A, Solanki S (2024). Blended RAG: improving RAG (retriever-augmented generation) accuracy with semantic search and hybrid query-based retrievers.

[46] Jeong S, Baek J, Cho S, Hwang S, Park J (2024). Adaptive-RAG: learning to adapt retrieval-augmented large language models through question complexity. https://arxiv.org/abs/2403.14403.

