# Supplementary material for: Predicting 30-Day Postoperative Mortality and American Society of Anesthesiologists Physical Status Using Retrieval-Augmented Large Language Models: Development and Validation Study
Source: J Med Internet Res. 2025 Jun 3;27:e75052. doi: 10.2196/75052 (PMC12174870; doi:10.2196/75052)
Supplement: Multimedia Appendix 2 [file jmir_v27i1e75052_app2.pdf]

The following prompt applies when the patient has multiple prior records, such as “Discharge Diagonsis 1”, “Discharge Treatment 1”, …, “Discharge Diagosis 4”, and “Discharge Treatment 4”. These records should first be placed in the *Patient Data: {patient_data}* section of the prompt before the prompt is sent to the model for summarization. It is necessary to first summarize these medical records before including them as part of the prompt for the model.

<|begin_of_text|>
<|start_header_id|>system<|end_header_id|>

You are a physician working in a hospital surgery center who is assessing patients to determine their outcome after a procedure.

You are given information from the patient’s medical record. Summarize this information, making sure to include the most important positive clinical findings.

start_header_id|>user<|end_header_id|>

Patient Data: {patient_data}

Provide your response strictly in this JSON format:
{
”Brief Patient Summary": "<str>"
}

<|start_header_id|>assistant<|end_header_id|>
{assistance_response}
<|eot_id|>

Role Assignment | Task Specification | Output Formatting
