# Supplementary material for: Predicting 30-Day Postoperative Mortality and American Society of Anesthesiologists Physical Status Using Retrieval-Augmented Large Language Models: Development and Validation Study
Source: J Med Internet Res. 2025 Jun 3;27:e75052. doi: 10.2196/75052 (PMC12174870; doi:10.2196/75052)
Supplement: Multimedia Appendix 5 [file jmir_v27i1e75052_app5.pdf]

Comparison of Embedding models for Clinical Risk Prediction

Embedding models play a crucial role in retrieval-augmented generation (RAG) frameworks by transforming unstructured clinical text into meaningful vector representations. The choice of embedding model can significantly impact the performance of downstream tasks, including mortality prediction and ASA classification. To assess the effect of different embedding models, we compared MedEmbed [1] and PubMedBERT [2] under identical experimental conditions, evaluating their impact on predictive accuracy and clinical relevance.

To ensure a fair comparison, both MedEmbed and PubMedBERT were evaluated under the same hyperparameter settings (temperature = 0.1, top_p = 0.1). Additionally, we assessed the models' performance across various retrieval top-k values (6, 8, 12). Performance was assessed using:

- AUPRC as the primary metric for mortality prediction, given its robustness in imbalanced datasets.
- Micro F1 Score as the primary metric for ASA classification, ensuring balanced evaluation across all classes.
- Recall (sensitivity) was also examined, as it is critical in clinical decision-making, where minimizing false negatives is essential.

Mortality Prediction Task

MedEmbed demonstrated superior performance in mortality prediction, achieving an AUPRC of 0.4733 (95% CI 0.4674 - 0.4792) at retrieval_top_k = 12, outperforming PubMedBERT's best AUPRC of 0.4249 (95% CI 0.4151 - 0.4347); see Figure S1.

Furthermore, MedEmbed exhibited higher recall, reaching 0.7917 (95% CI 0.7795 - 0.8039) at retrieval_top_k = 12, compared to PubMedBERT's best recall of 0.7083 (95% CI 0.6963 - 0.7203) (Multimedia Appendix 3). This highlights MedEmbed's advantage in identifying high-risk patients, which is particularly valuable for clinical applications.

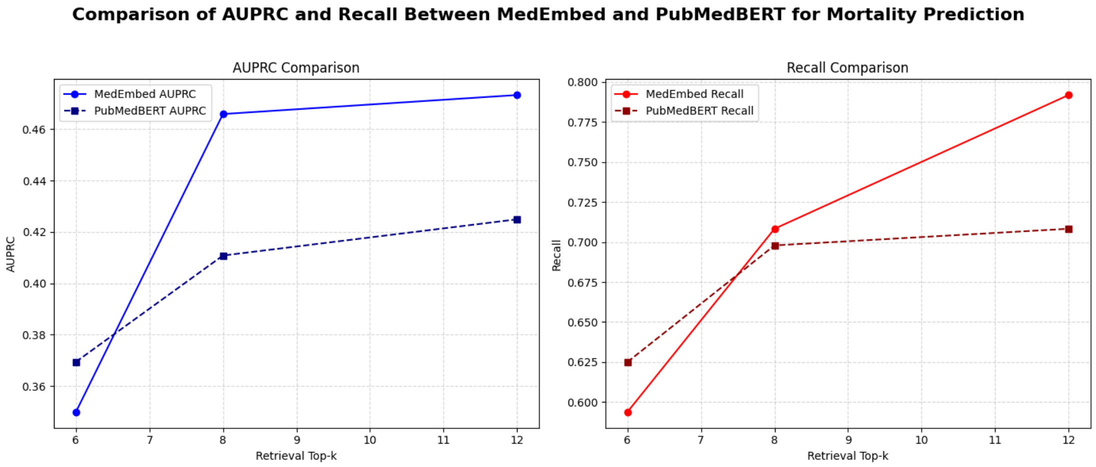

Figure S1. Comparison of AUPRC and Recall Between MedEmbed and PubMedBERT for Mortality Prediction.

ASA Classification Task

For ASA classification, MedEmbed continued to outperform PubMedBERT, achieving the highest Micro F1 Score of 0.6307 (95% CI 0.6127 - 0.6540) at retrieval_top_k = 6, whereas PubMedBERT's best performance was 0.5958 (95% CI 0.5671 - 0.6188); see Figure S2. Similarly, in terms of recall, MedEmbed attained 0.6307 (95% CI 0.6023 - 0.6591), surpassing PubMedBERT's best recall of 0.5958 (95% CI 0.5660 - 0.6256) (Multimedia Appendix 3). These results indicate that MedEmbed provides a more reliable classification of patient ASA status, crucial for perioperative risk stratification.

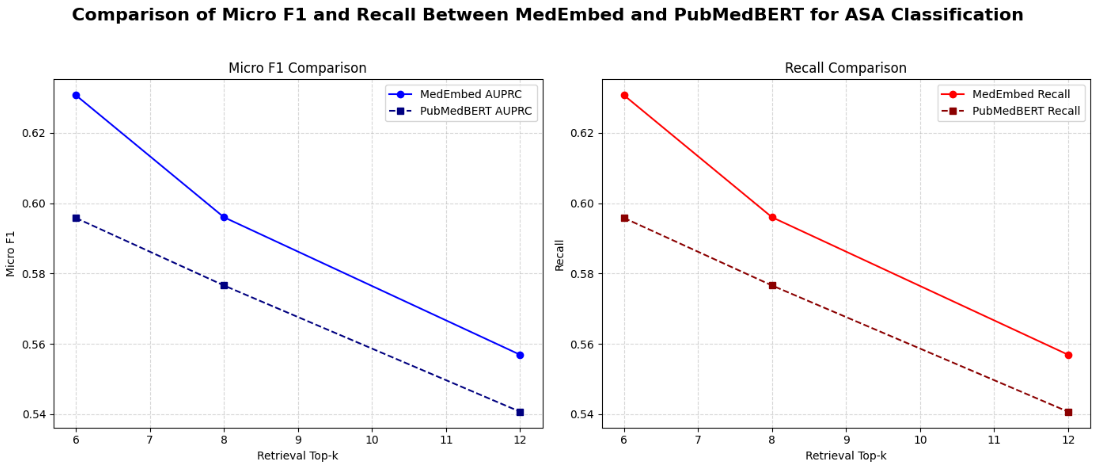

Figure S2. Comparison of Micro F1 Score and Recall Between MedEmbed and PubMedBERT for ASA Classification.

Overall, MedEmbed consistently outperformed PubMedBERT in both mortality prediction and ASA classification, particularly in improving recall, a key metric for clinical applications. Given these findings, we adopted MedEmbed as the embedding model for all subsequent experiments, ensuring optimal model performance in our study.

Impact of Chuck Size on Clinical Risk Prediction

Chunk size is a crucial parameter in retrieval-augmented generation (RAG) frameworks, as it determines how clinical text is segmented before embedding and retrieval. An optimal chunk size balances information granularity and retrieval efficiency, which can significantly influence model performance. To identify the best chunk size for clinical risk prediction, we systematically evaluated different chunk size settings across mortality prediction and ASA classification tasks.

We conducted experiments with various chunk sizes (150, 200, 250, 300, and 350) using MedEmbed as the embedding model. Performance was assessed using:

- AUPRC for mortality prediction, given its effectiveness in imbalanced datasets.
- Micro F1 Score for ASA classification, ensuring fair evaluation across all ASA classes.

Each chunk size was tested across different retrieval_top_k values (6, 8, 12) under a fixed temperature of 0.1 and top_p of 0.1 to determine the most effective configuration.

Mortality Prediction Task

As shown in Figure S3, when performing mortality prediction, setting chunk size to 250 resulted in the highest AUPRC of 0.4659 (95% CI 0.4628 - 0.4690) at retrieval_top_k = 8 and 0.4733 (95% CI 0.4674 - 0.4792) at retrieval_top_k = 12. However, when retrieval_top_k was 6, the optimal chunk size was 200, achieving an AUPRC of 0.4302 (95% CI 0.4218 - 0.4386).

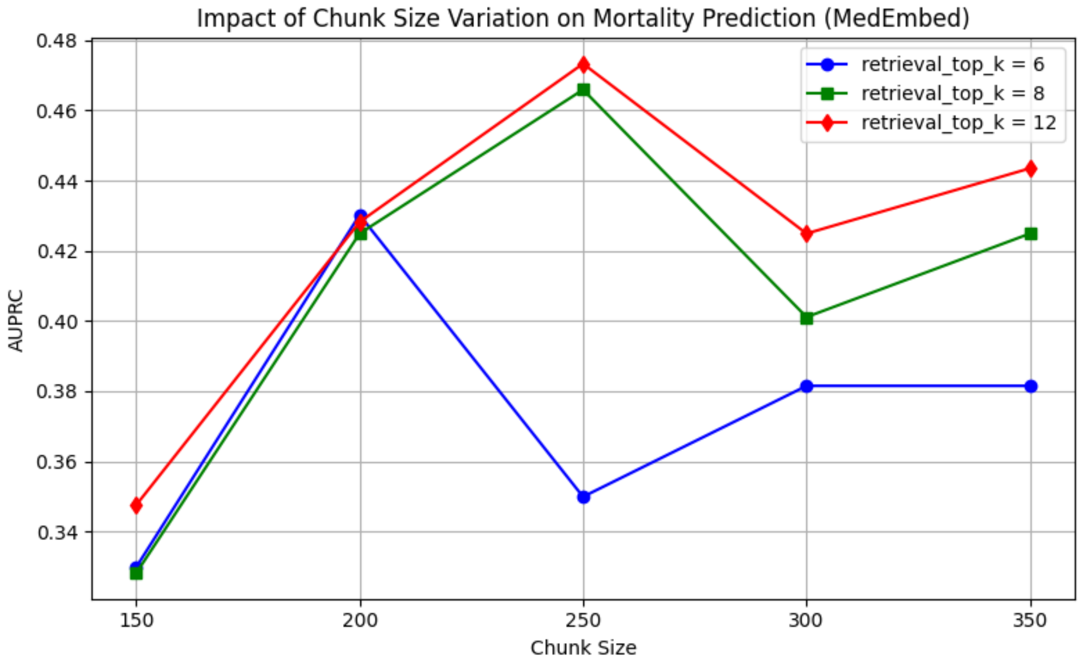

Figure S3. Impact of Chuck Size Variation on Mortality Prediction.

ASA Classification Task

For ASA classification (Figure S4), chunk size 250 also demonstrated strong performance, yielding the highest Micro F1 Score of 0.6307 (95% CI 0.6127 - 0.6540) at retrieval top_k = 6 and 0.5960 (95% CI 0.5900 - 0.6168) at retrieval top_k = 8. However, for retrieval top_k = 12, a larger chunk size of 350 produced the best Micro F1 Score of 0.5869 (95% CI 0.5696 - 0.6128).

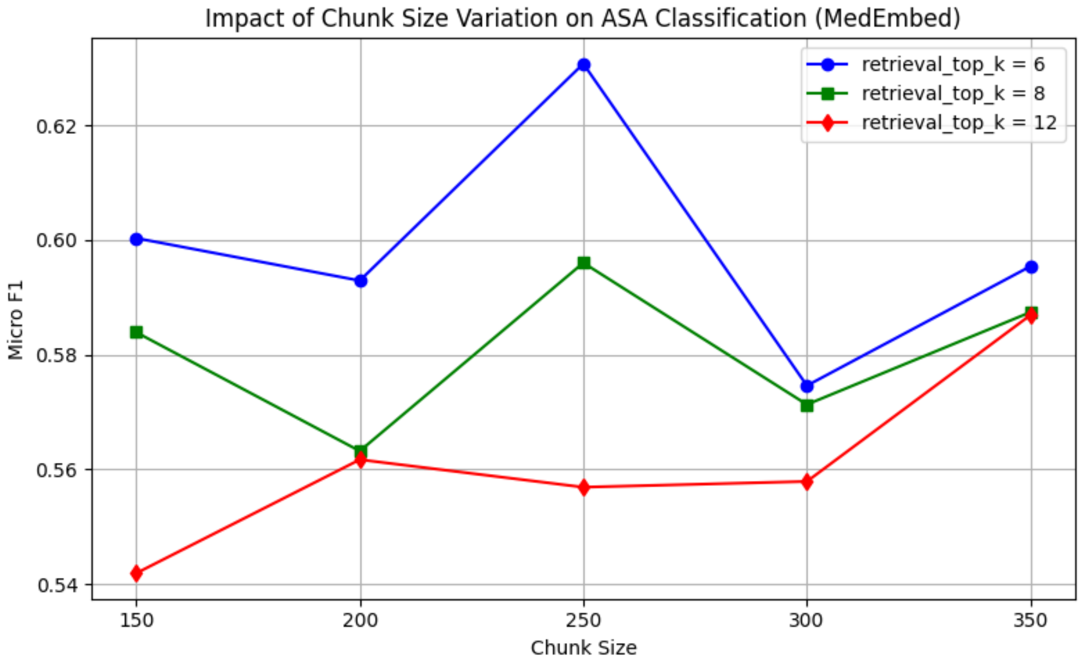

Figure S4. Impact of Chuck Size Variation on ASA Classification.

These results highlight the critical role of chunk size in retrieval-based clinical prediction models. While chunk size = 250 was generally optimal across tasks, performance variations at different retrieval top_k indicate that chunk size tuning should be task-dependent. Given these findings, chunk size = 250 was adopted for subsequent experiments, ensuring robust performance across both mortality prediction and ASA classification.

Impact of Few-Shot Learning on Clinical Risk Prediction

Few-shot learning has been widely recognized for its ability to enhance the performance of large language models by providing contextual examples. In clinical risk prediction, selecting an optimal number of few-shot examples is crucial for maximizing predictive accuracy [3]. This section evaluates the impact of incorporating different few-shot settings on mortality prediction and ASA classification.

To investigate the effectiveness of few-shot learning, we tested models under few-shot settings and compared them against the baseline zero-shot (without few-shot) configuration. To ensure a comprehensive evaluation, we explored three different hyperparameter configurations for temperature and top_p: (0.001, 0.001), (0.1, 0.1), and (1, 0.5). While retrieval top_k was initially varied across 6, 8, and 12, we present results using only retrieval top_k = 8, as it yielded the highest performance. This approach allows for a focused comparison of how different sampling strategies influence few-shot effectiveness while maintaining consistency in retrieval settings.

Mortality Prediction Task

In the mortality prediction task, the 9-shot setting achieved the highest AUPRC of 0.6536 (95% CI 0.6479 - 0.6593) under the hyperparameter configuration (temperature = 1, top_p = 0.5, retrieval top_k = 8), outperforming the 5-shot setting, which reached 0.6176 (95% CI 0.6034 - 0.6318) under the same conditions.; see Figure S5.

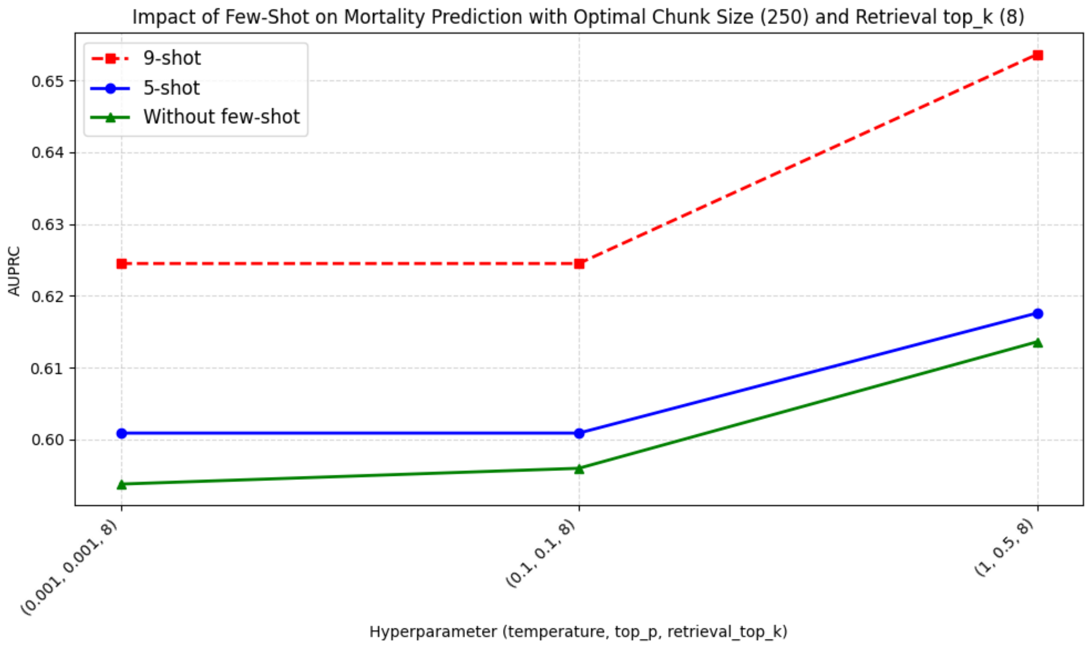

Figure S5. Impact of Few-Shot on Mortality Prediction. In the mortality prediction task, the 9-shot configuration was chosen to ensure that each ASA Class (1 to 5) included examples of patients who died within 30 days as well as those who survived beyond 30 days. However, in ASA Class 1, there were no patients in the dataset who met the 30-day mortality criterion, making it impossible to include a complete set of examples. To account for this limitation, the few-shot examples were randomly selected from the available cases in each ASA class. Consequently, we selected 9 examples for the few-shot setup instead of a full set of 10 examples.

ASA Classification Task

In the ASA classification task, few-shot learning significantly improved model performance. Under the hyperparameter setting of temp=0.001, top_p=0.001, and retrieval top_k=8, the highest Micro F1 Score of 0.8409 (95% CI 0.8238 - 0.8551) was achieved with few-shot prompting, whereas the zero-shot (without few-shot) configuration only reached 0.5839 (95% CI 0.5763 - 0.5884) under the same setting; see Figure S6. This substantial performance gap demonstrates the effectiveness of providing structured contextual examples for complex classification tasks.

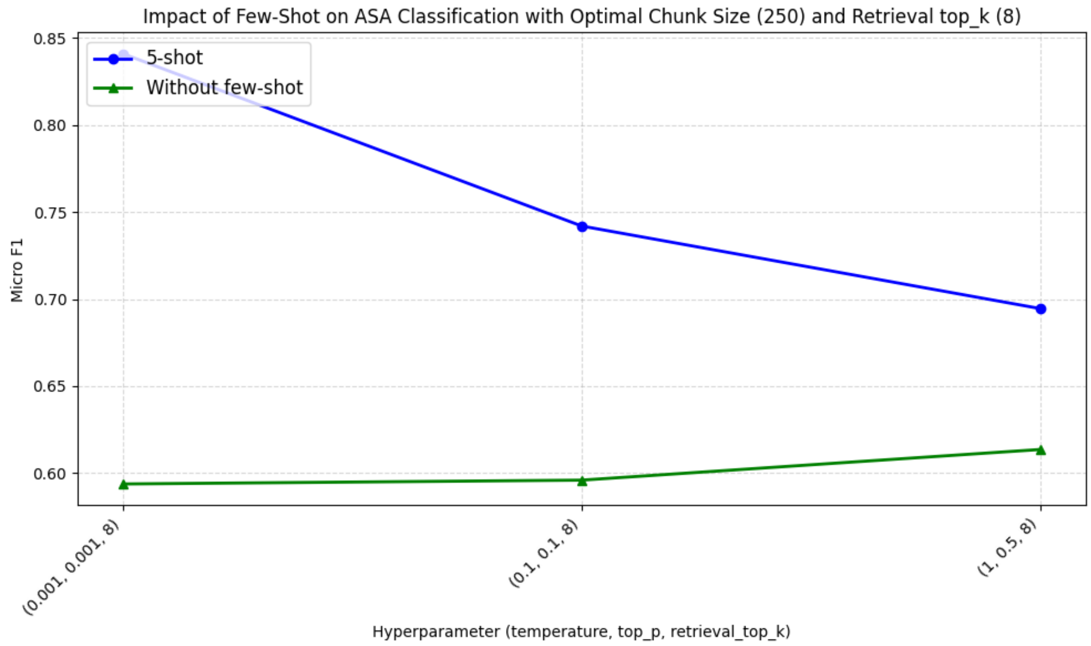

Figure S6. Effect of Few-Shot on ASA Classification.

These results indicate that incorporating few-shot examples generally improves model performance. However, the observed fluctuations in performance for different few-shot settings emphasize the necessity of careful example selection. Future work should explore adaptive selection strategies to maximize the effectiveness of few-shot learning in clinical applications.

Reference

1. Abhinand P. MedEmbed: Fine-Tuned Embedding Models for *Medical / Clinical IR*. Medium; 2023. Available from: https://abhinand05.medium.com/medembed-fine-tuned-embedding-models-for-medical-clinical-ir-5595b0937b58
2. Gu Y, Tinn R, Cheng H, et al. Domain-Specific Language Model Pretraining for Biomedical Natural Language Processing. ACM Trans Comput Healthc. 2022;3(1):2:1-2:23. Available from: https://doi.org/10.1145/3458754
3. Chung P, Fong CT, Walters AM, Aghaeepour N, Yetisgen M, O'Reilly-Shah VN. Large language model capabilities in perioperative risk prediction and prognostication. *JAMA Surg*; 2024;159(8):928-937. doi:10.1001/jamasurg.2024.1621.
