# Supplementary material for: Predicting 30-Day Postoperative Mortality and American Society of Anesthesiologists Physical Status Using Retrieval-Augmented Large Language Models: Development and Validation Study
Source: J Med Internet Res. 2025 Jun 3;27:e75052. doi: 10.2196/75052 (PMC12174870; doi:10.2196/75052)
Supplement: Multimedia Appendix 6 [file jmir_v27i1e75052_app6.pdf]

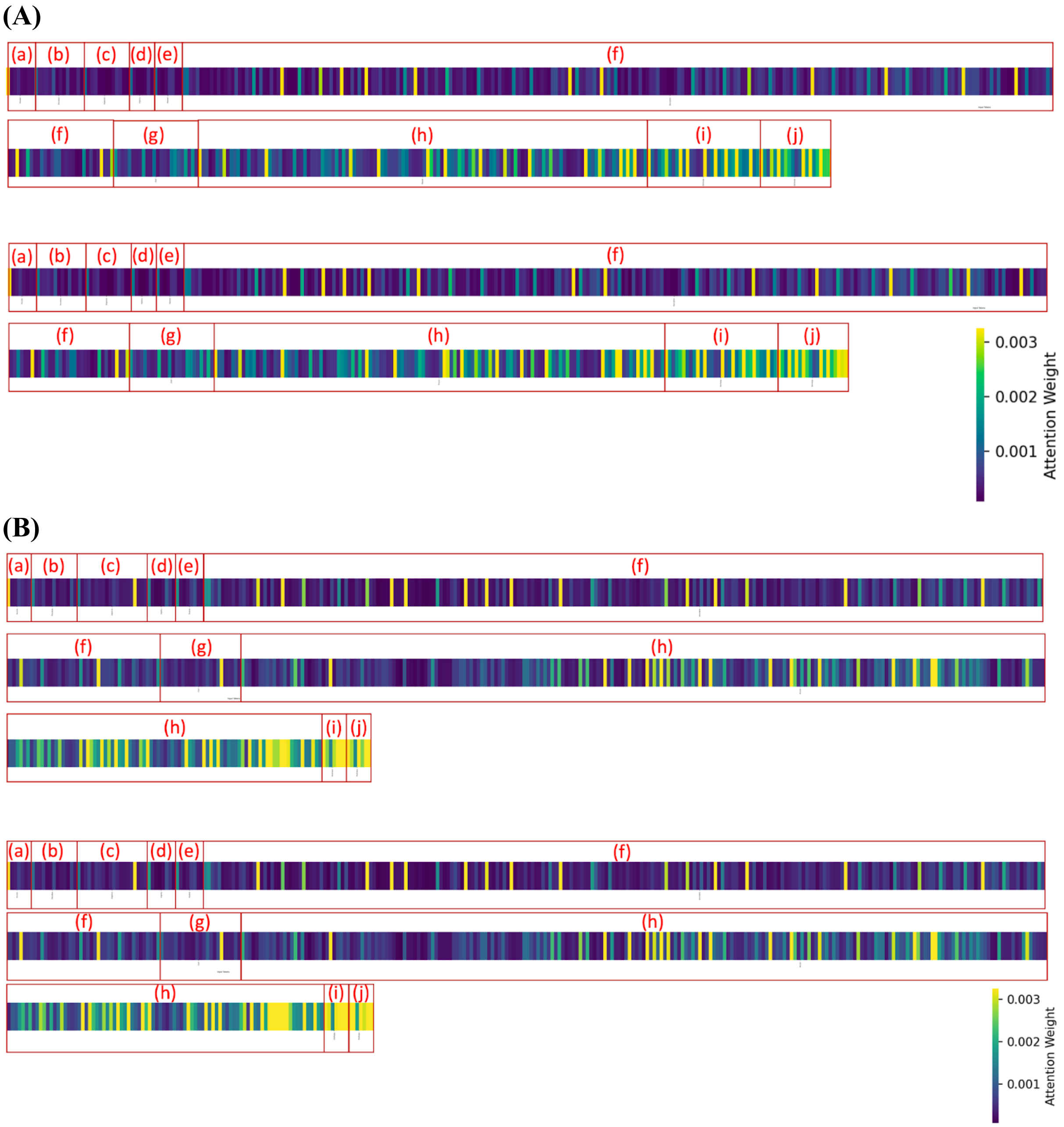

Figure. Attention Weight Visualization for LLaMA-3.1 + RAG

This figure displays four horizontal heatmaps of normalized attention weights from the model's final output token onto each input token, grouped by clinical sections:

(a) Provider, (b) Procedure, (c) Diagnosis, (d) Urgency Level, (e) Planned Anesthesia, (f) Description, (g) Chief Complaint, (h) Present Illness, (i) Discharge Diagnosis, (j) Discharge Treatment.

- **Panel A upper (Patient 1, ASA I):** Highest attention on present illness (h), discharge diagnosis (i), and discharge treatment (j), suggesting downstream outcomes inform ASA assignment.
- **Panel A lower (Patient 1, Low Mortality Risk):** Concentrated on present illness (h) and discharge diagnosis (i), emphasizing acute clinical trajectory for mortality prediction.

- **Panel B upper (Patient 2, ASA V):** Focused almost exclusively on present illness (h), indicating reliance on recent clinical course for high ASA status.
- **Panel B lower (Patient 2, High Mortality Risk):** Attention sharply on present illness (h) with minimal discharge-related weighting, highlighting in-hospital progression as the driver of risk.

Color intensity—from purple (near-zero attention) through green to yellow (strong attention)—is clipped at the 90th percentile to prevent outliers from obscuring key patterns. These maps consistently show that **present illness**, along with **discharge diagnosis** and **discharge treatment**, are the primary sections the model uses when stratifying ASA-PS and 30-day mortality risk, underscoring a clinically intuitive focus that may enhance transparency and trust in perioperative decision support.
