# Supplementary material for: Predicting 30-Day Postoperative Mortality and American Society of Anesthesiologists Physical Status Using Retrieval-Augmented Large Language Models: Development and Validation Study
Source: J Med Internet Res. 2025 Jun 3;27:e75052. doi: 10.2196/75052 (PMC12174870; doi:10.2196/75052)
Supplement: Multimedia Appendix 8 [file jmir_v27i1e75052_app8.pdf]

A.

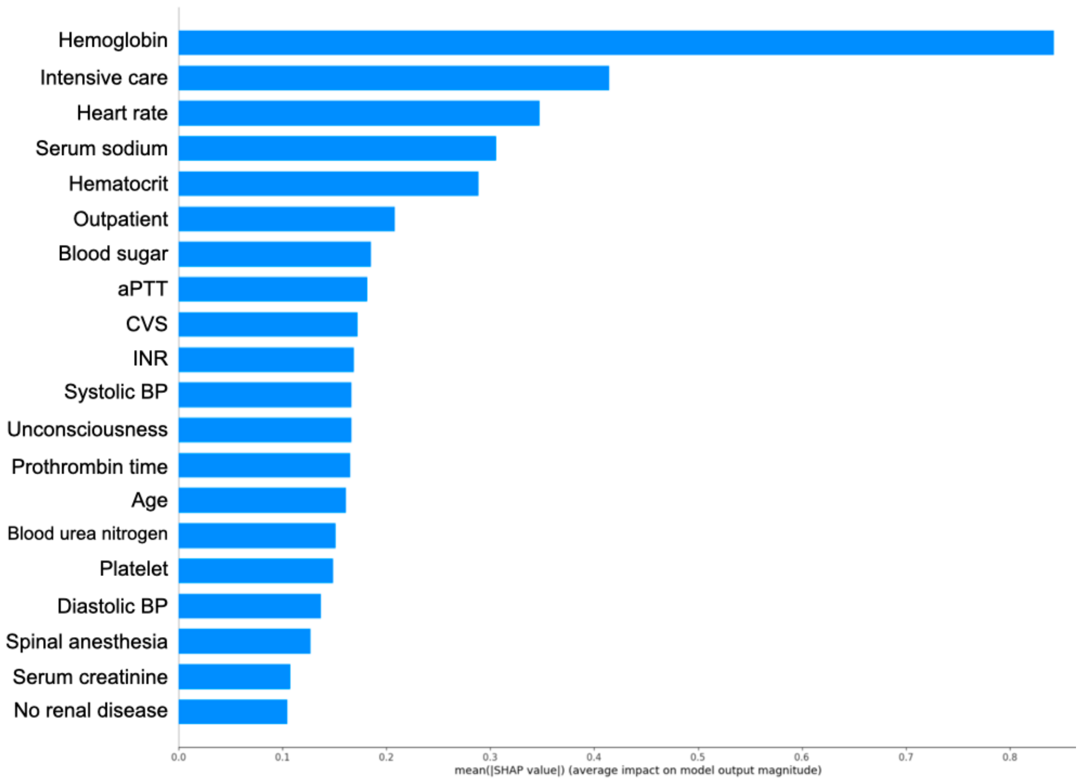

Hemoglobin
Intensive care
Heart rate
Serum sodium
Hematocrit
Outpatient
Blood sugar
aPTT
CVS
INR
Systolic BP
Unconsciousness
Prothrombin time
Age
Blood urea nitrogen
Platelet
Diastolic BP
Spinal anesthesia
Serum creatinine
No renal disease
0.0
0.1
0.2
0.3
0.4
0.5
0.6
0.7
0.8
mean(|SHAP value|) (average impact on model output magnitude)

B.

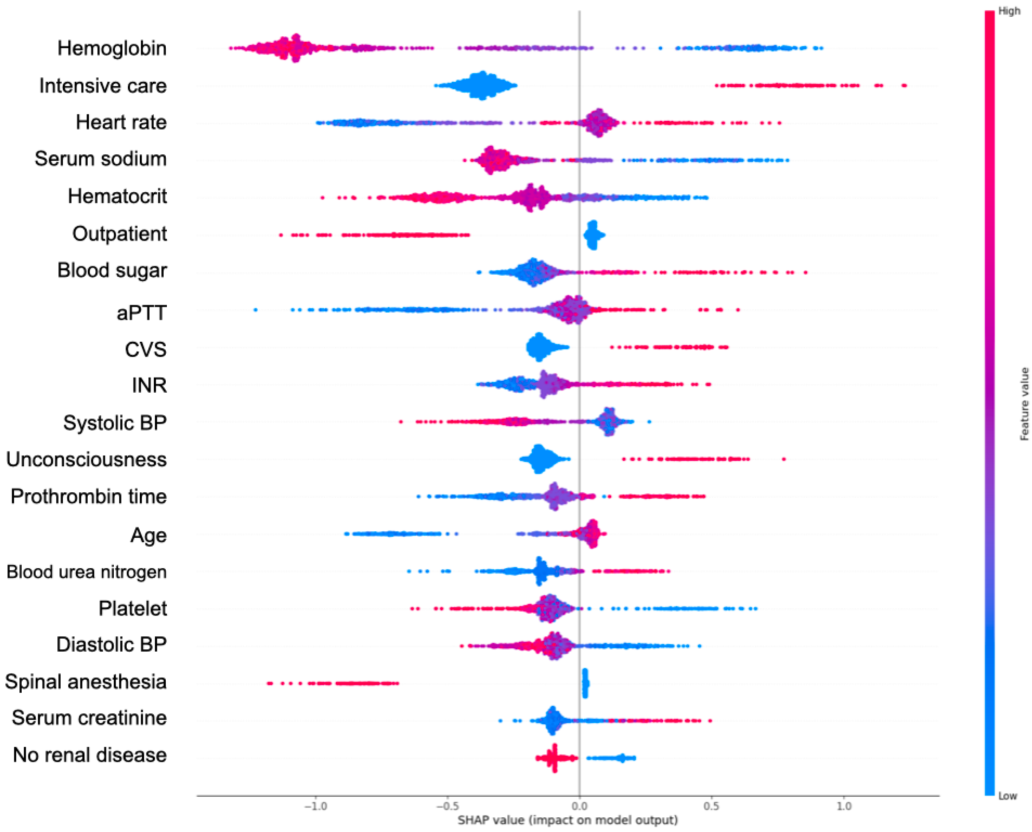

Hemoglobin
Intensive care
Heart rate
Serum sodium
Hematocrit
Outpatient
Blood sugar
aPTT
CVS
INR
Systolic BP
Unconsciousness
Prothrombin time
Age
Blood urea nitrogen
Platelet
Diastolic BP
Spinal anesthesia
Serum creatinine
No renal disease
−1.0
−0.5
0.0
0.5
1.0
SHAP value (impact on model output)
High
Feature value
Low

Figure 1. Global Explainability of the XGBoost Mortality Prediction Model using SHAP. CVS, cardiovascular surgery; CVA, cerebrovascular accident; BP, blood pressure; BMI, body mass index; aPTT, activated partial thromboplastin time.

A. Mean absolute SHAP value bar plot for the top 20 features, calculated over 1,000 randomly selected training samples. Hemoglobin is the most influential predictor, lower values markedly increasing mortality risk, while intensive care and heart rate also rank highly.

B. SHAP summary dot plot for the same 20 features. Each dot represents a patient, with the x-axis showing the SHAP value (effect on model output) and color indicating the normalized feature value (red = high, blue = low). The horizontal spread visualizes direction and magnitude of impact (e.g., low hemoglobin—blue dots on the right—increases risk), nonlinear effects appear as violin-like structures, and categorical clusters reveal feature-specific risk associations. Density of points at each SHAP value, reflecting variability across patients.

Top predictors are **anemia**, **intensive care**, **tachycardia**, and **serum sodium**—align with established perioperative mortality risk factors, confirming that the model captures clinically valid patterns.

A.

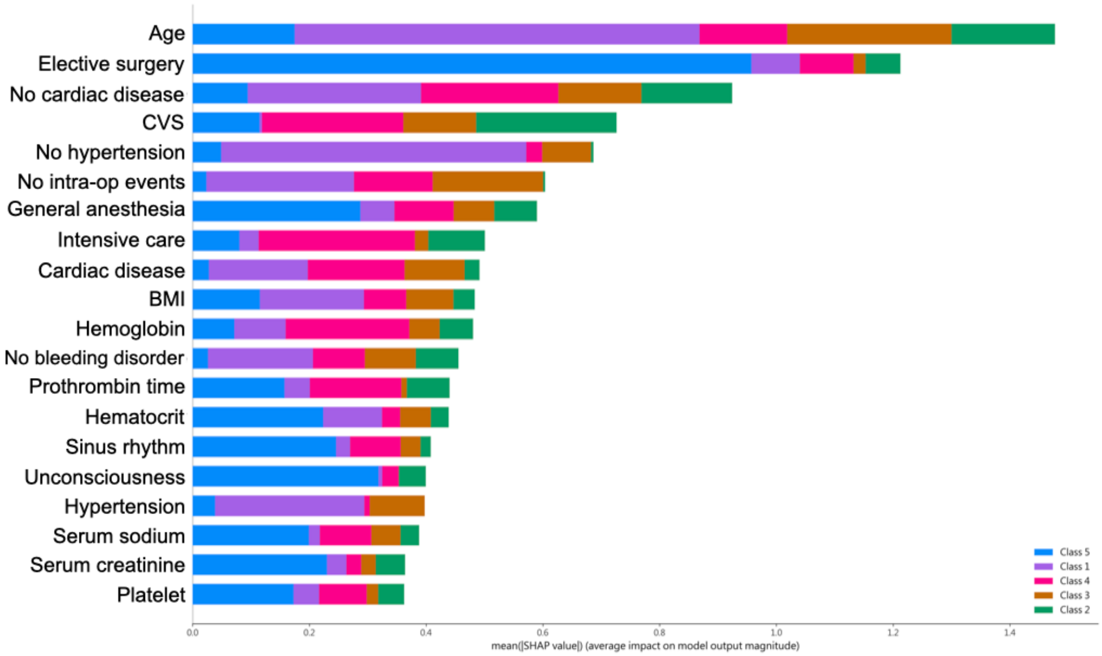

B-1. ASA-PS class 1

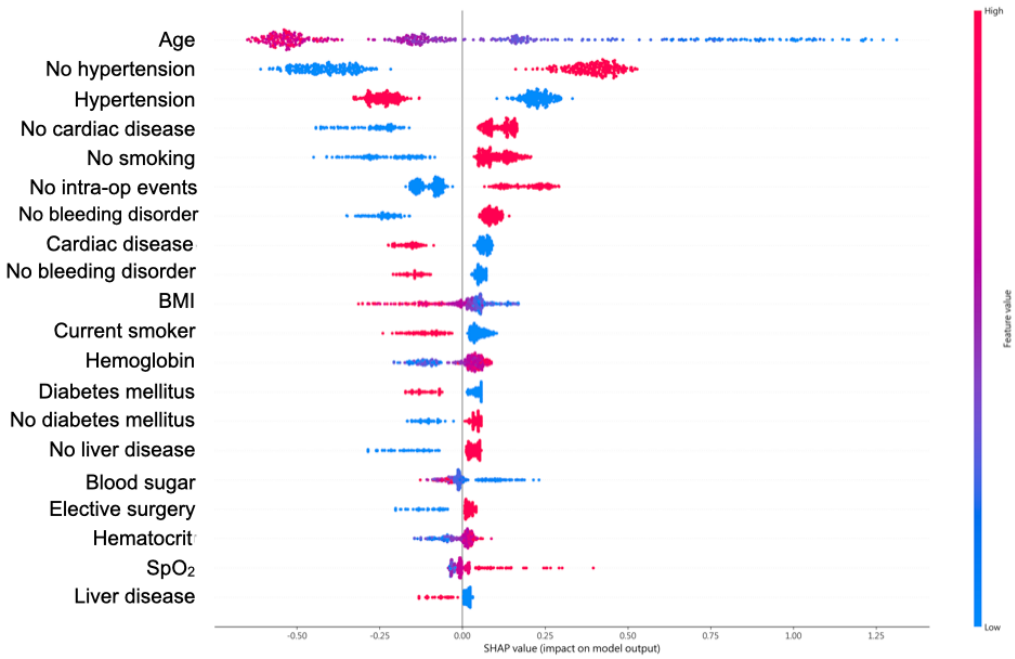

B-2. ASA-PS class 2

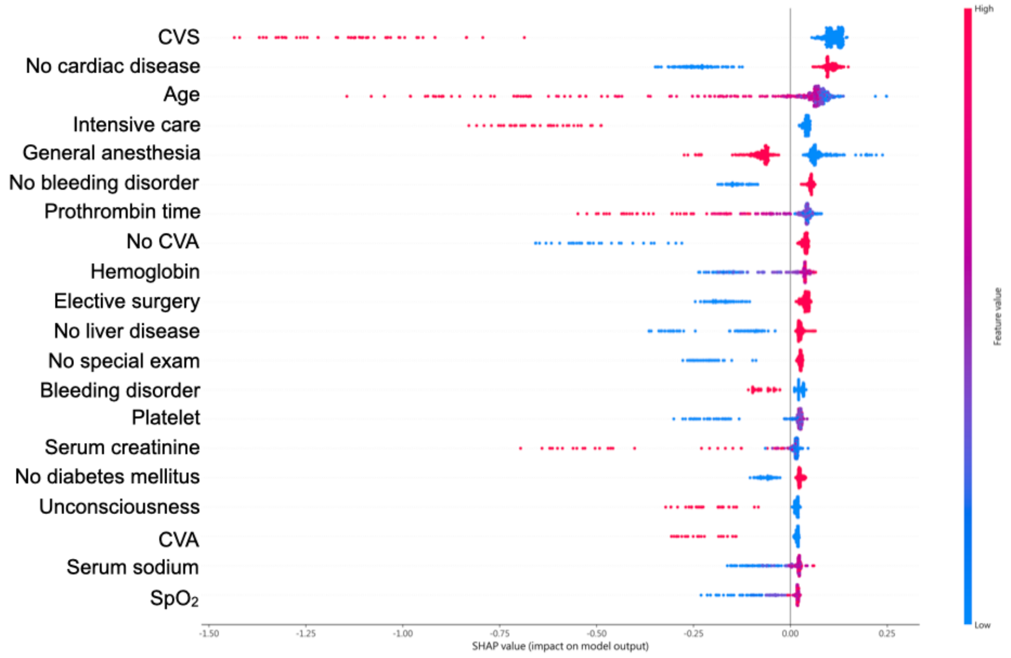

B-3. ASA-PS class 3

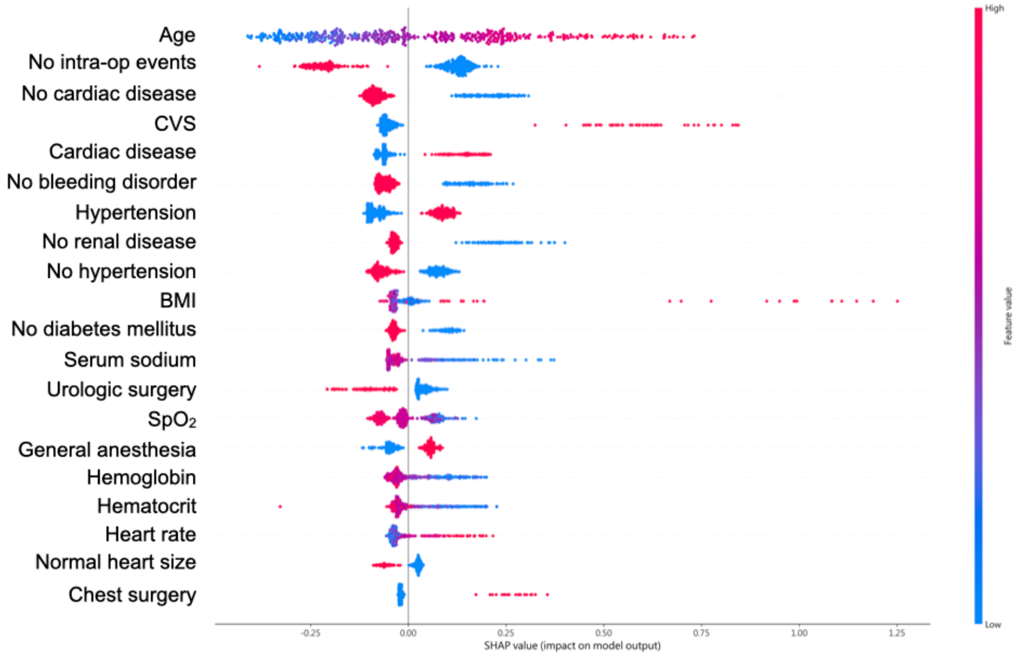

B-4. ASA-PS class 4

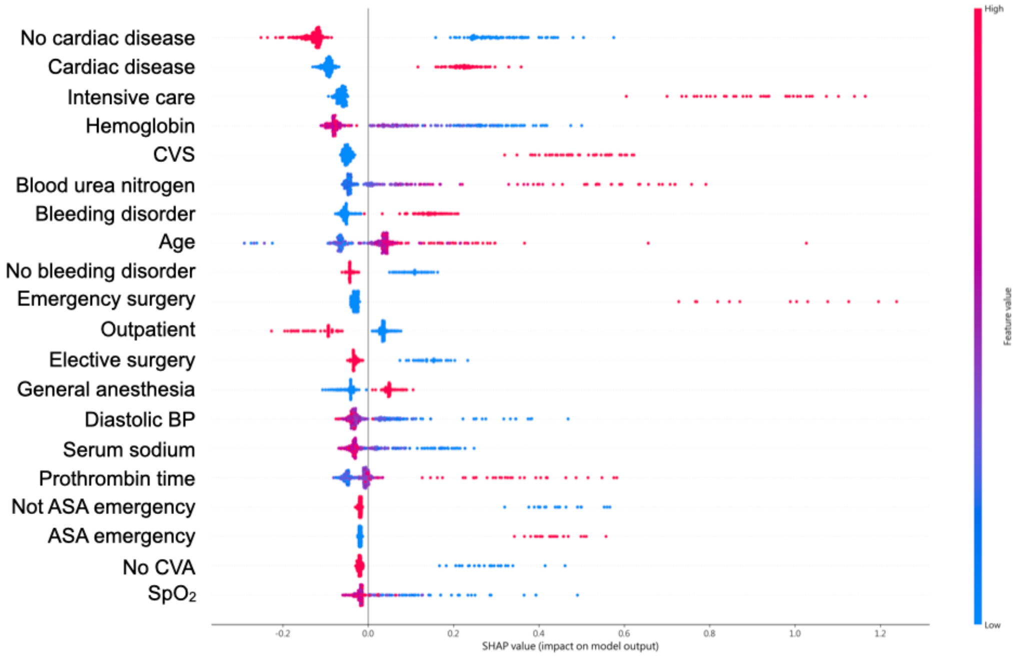

B-5. ASA-PS class 5

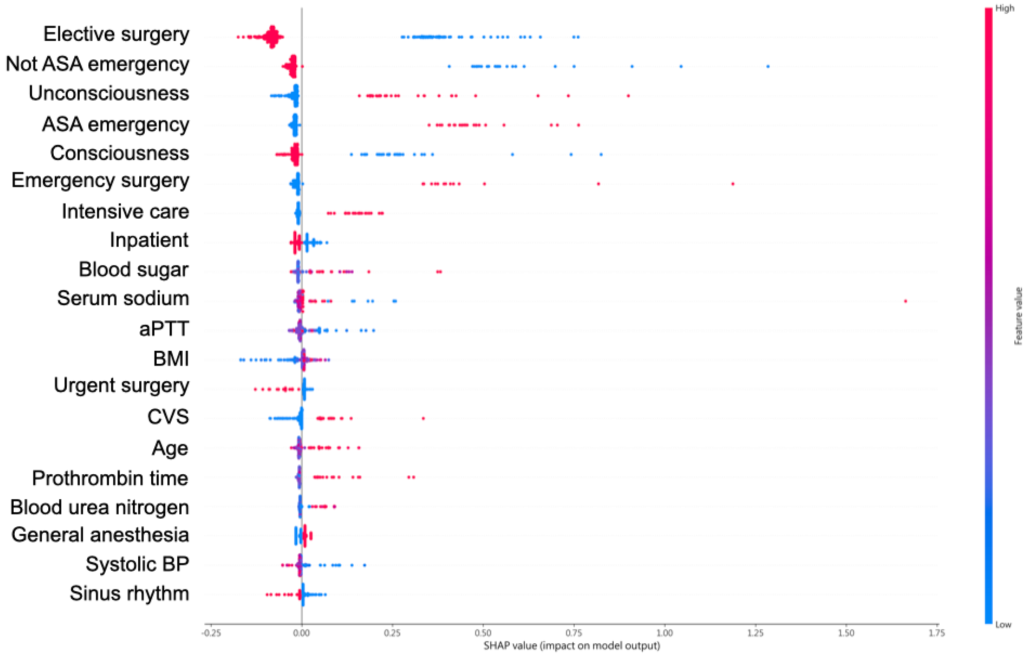

Figure 2. Global Explainability of the XGBoost ASA-PS classification Model using SHAP. CVS, cardiovascular surgery; CVA, cerebrovascular accident; BP, blood pressure; BMI, body mass index; aPTT, activated partial thromboplastin time.

A. Global SHAP bar plot showing the top 20 features ranked by their mean absolute SHAP value across the entire dataset (all classes combined). Higher bars indicate greater overall impact on model predictions.

B1–B5. Class-specific SHAP summary (beeswarm) plots, one for each ASA class. Each dot represents a patient, with the x-axis showing the SHAP value (effect on model output) and color indicating the normalized feature value (red = high, blue = low). The horizontal spread visualizes direction and magnitude of impact (e.g., low hemoglobin—blue dots on the right—increases risk), nonlinear effects appear as violin-like structures, and categorical clusters reveal feature-specific risk associations. Density of points at each SHAP value, reflecting variability across patients.

These visualizations enable comparison of global feature importance (Figure A) and detailed, class-level explainability (Figures B1–B5), highlighting which clinical factors most strongly drive ASA-PS assignments. Across all five ASA classes, **age**, **elective surgery**, and **cardiac disease** emerged as the strongest predictors in the global SHAP bar plot, followed closely by **surgical department (notably cardiovascular surgery)** and **hypertension status**. The class-specific beeswarm plots further show that **higher age**, **emergency surgery**, and **cardiac disease presence** consistently drive up the predicted ASA-PS score, while features such as **no intra-operative events** tend to lower it.
