# Supplementary material for: Predicting 30-Day Postoperative Mortality and American Society of Anesthesiologists Physical Status Using Retrieval-Augmented Large Language Models: Development and Validation Study
Source: J Med Internet Res. 2025 Jun 3;27:e75052. doi: 10.2196/75052 (PMC12174870; doi:10.2196/75052)
Supplement: Multimedia Appendix 9 [file jmir_v27i1e75052_app9.pdf]

Mortality Prediction – Statistical Significance

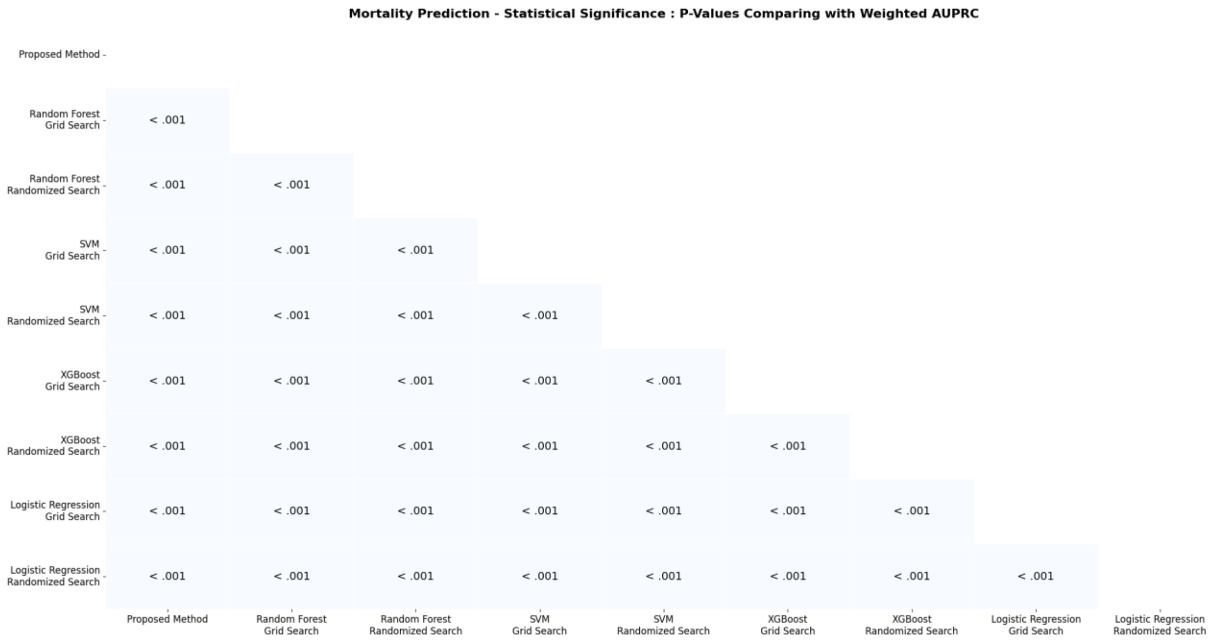

P-values comparing across different models with weighted AUPRC for Mortality Prediction. *P*-values are computed by resampling the test set 1000 times and measuring the difference in Weighted AUPRC between the proposed method and other models. *P*-values smaller than 0.001 are reported as “< .001”.

ASA Classification– Statistical Significance

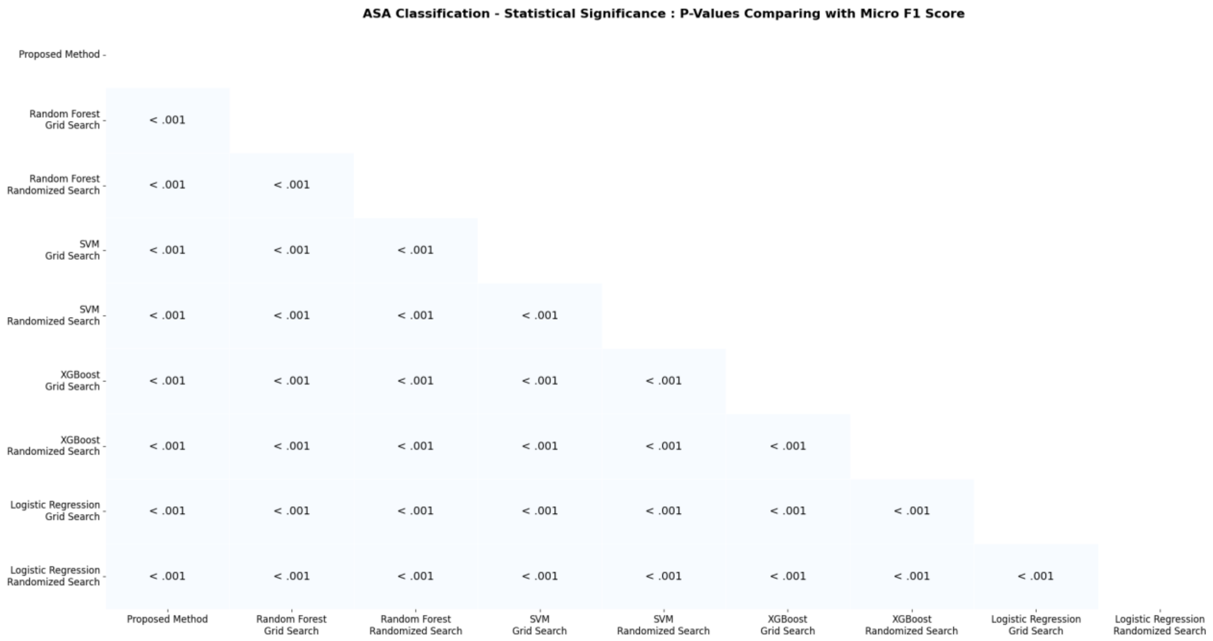

P-values comparing across different models with Micro F1 Score for ASA Classification. *P*-values are computed by resampling the test set 1000 times and measuring the difference in Micro F1 Score between the proposed method and other models. *P*-values smaller than 0.001 are reported as “< .001”.

Reference

1. Boyd K, Eng KH, Page CD. Area under the precision-recall curve: point estimates and confidence intervals. In: Blockeel H, Kersting K, Nijssen S, Železný F, editors. *Machine Learning and Knowledge Discovery in Databases*. ECML PKDD 2013. Lecture Notes in Computer Science, vol 8190. Berlin, Heidelberg: Springer; 2013. p. 451-466. doi: 10.1007/978-3-642-40994-3_29.
