# Supplementary material for: Predicting 30-Day Postoperative Mortality and American Society of Anesthesiologists Physical Status Using Retrieval-Augmented Large Language Models: Development and Validation Study
Source: J Med Internet Res. 2025 Jun 3;27:e75052. doi: 10.2196/75052 (PMC12174870; doi:10.2196/75052)
Supplement: Multimedia Appendix 11 [file jmir_v27i1e75052_app11.pdf]

Confusion Matrix for Mortality Prediction

Machine Learning method

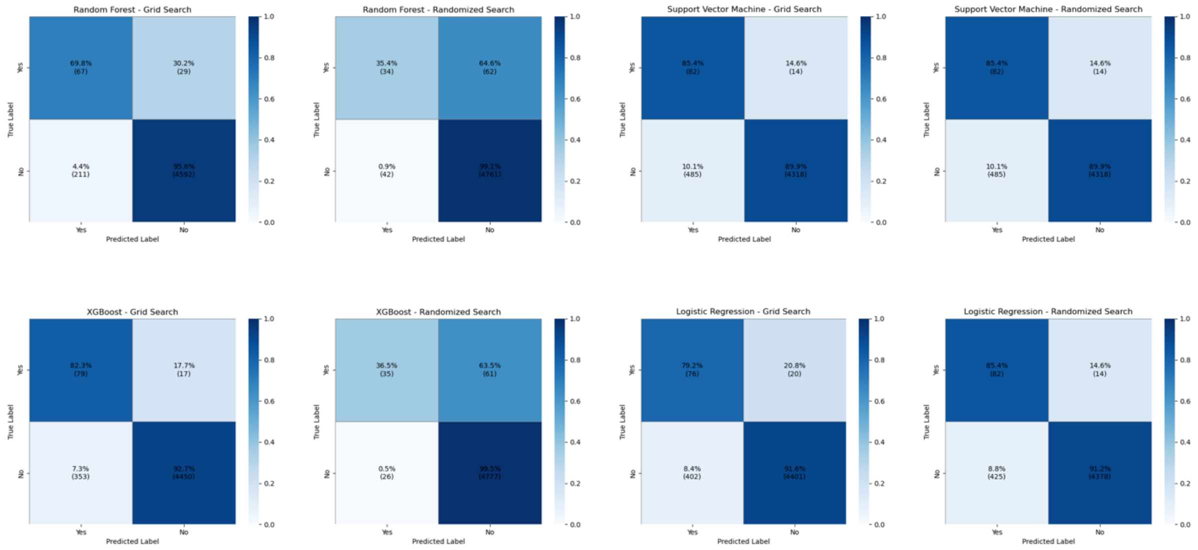

Figure 4.1. Confusion matrix for mortality prediction: using machine learning models.

Comparing different LLaMA versions with 5/9-shot

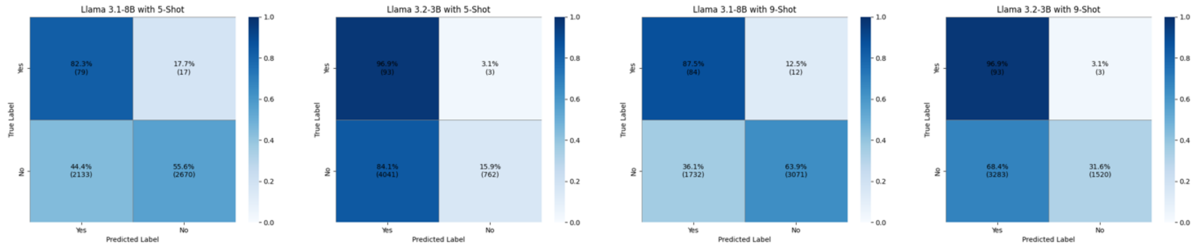

Figure 4.2. Confusion matrix for mortality prediction: comparison of different LLaMA versions with 5-Shot and 9-Shot settings.

Quantized Llama 3.1-8B (temperature, top_p, retrieval_top_k, chunk_size)

Without RAG and without few-shot

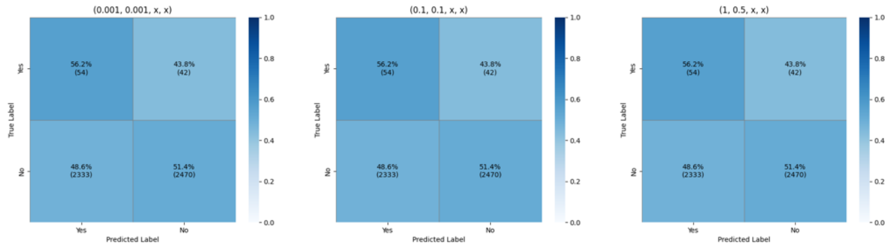

Figure 4.3. Confusion matrix for mortality prediction: without RAG, without few-shot.

Without RAG and with 5-shot

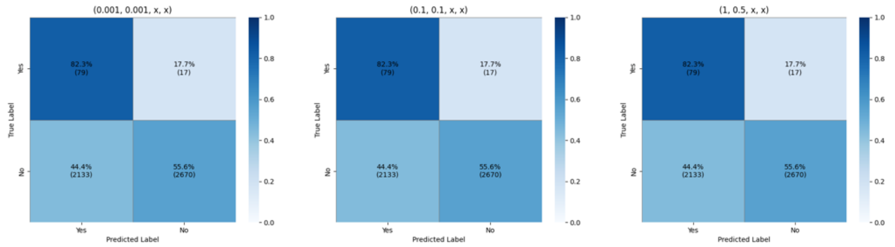

Figure 4.4. Confusion matrix for mortality prediction: without RAG, with 5-shot.

Confusion Matrix for Mortality Prediction

Quantized Llama 3.1-8B (temperature, top_p, retrieval_top_k, chunk_size)

Without RAG and with 9-shot

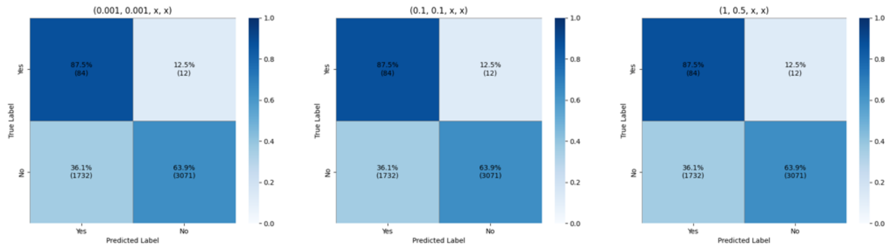

Figure 4.5. Confusion matrix for mortality prediction: without RAG, with 9-shot.

With RAG and without few-shot (MedEmbed)

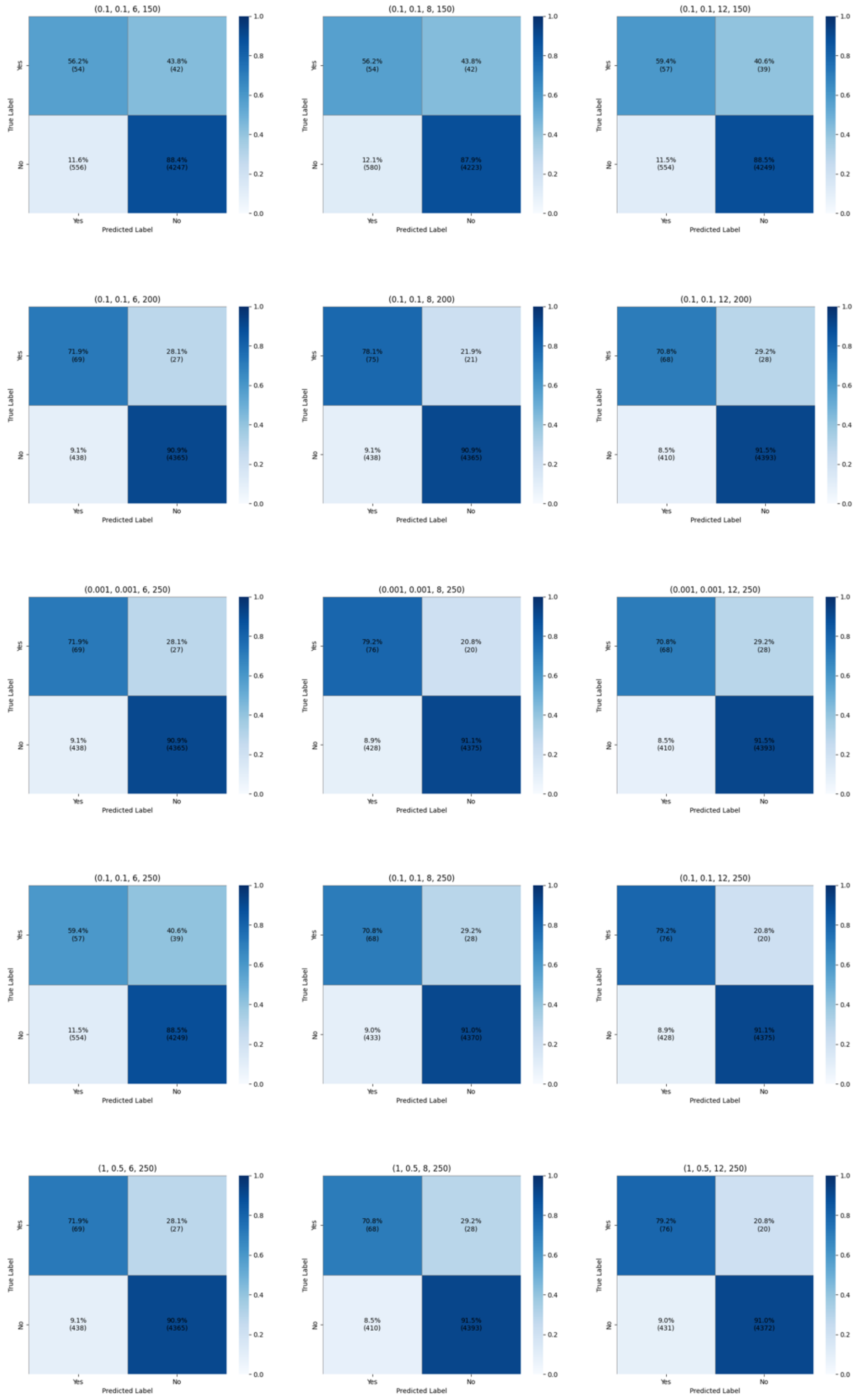

Figure 4.6. Confusion matrix for mortality prediction: with RAG, without few-shot (MedEmbed).

Confusion Matrix for Mortality Prediction

Quantized Llama 3.1-8B (temperature, top_p, retrieval_top_k, chunk_size)

With RAG and without few-shot (MedEmbed)

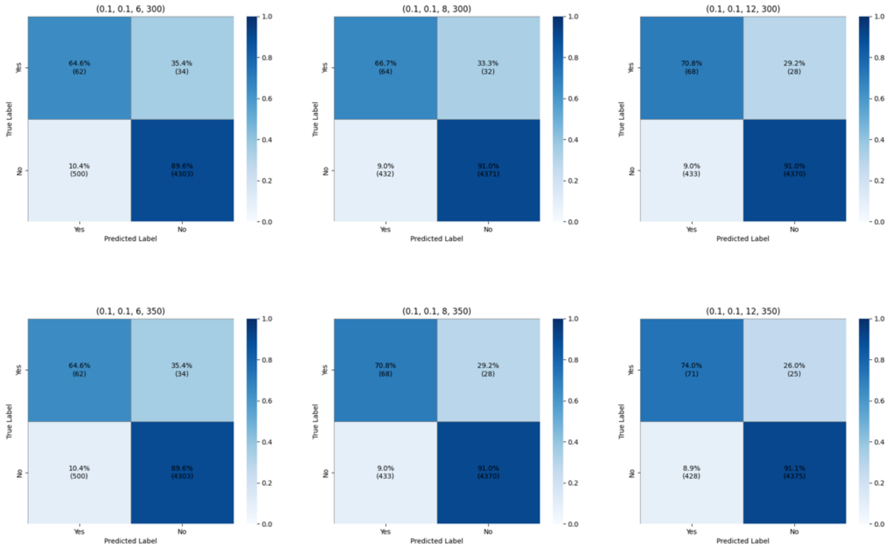

Figure 4.6-2. Confusion matrix for mortality prediction: with RAG, without few-shot (MedEmbed).

With RAG and without few-shot (pubmedbert)

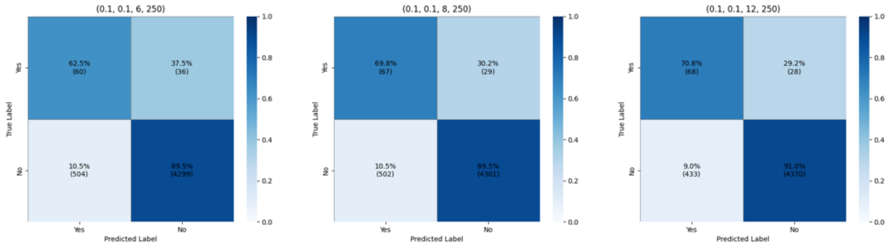

Figure 4.7. Confusion matrix for mortality prediction: with RAG, without few-shot (pubmedbert).

With RAG and With 5-shot (MedEmbed)

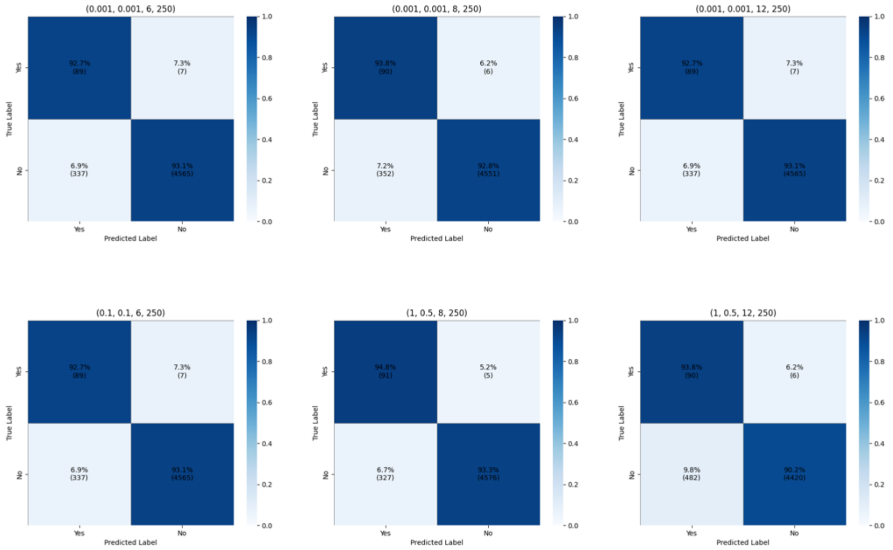

Figure 4.8. Confusion matrix for mortality prediction: with RAG, with 5-shot (MedEmbed).

Confusion Matrix for Mortality Prediction

Quantized Llama 3.1-8B (temperature, top_p, retrieval_top_k, chunk_size)

With RAG and with 5-shot (MedEmbed)

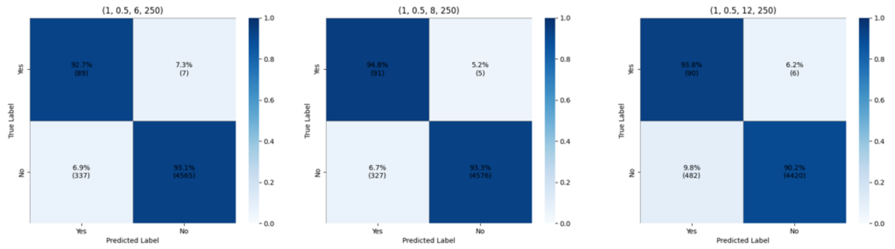

Figure 4.8-2. Confusion matrix for mortality prediction: with RAG, with 5-shot (MedEmbed).

With RAG and with 9-shot (MedEmbed)

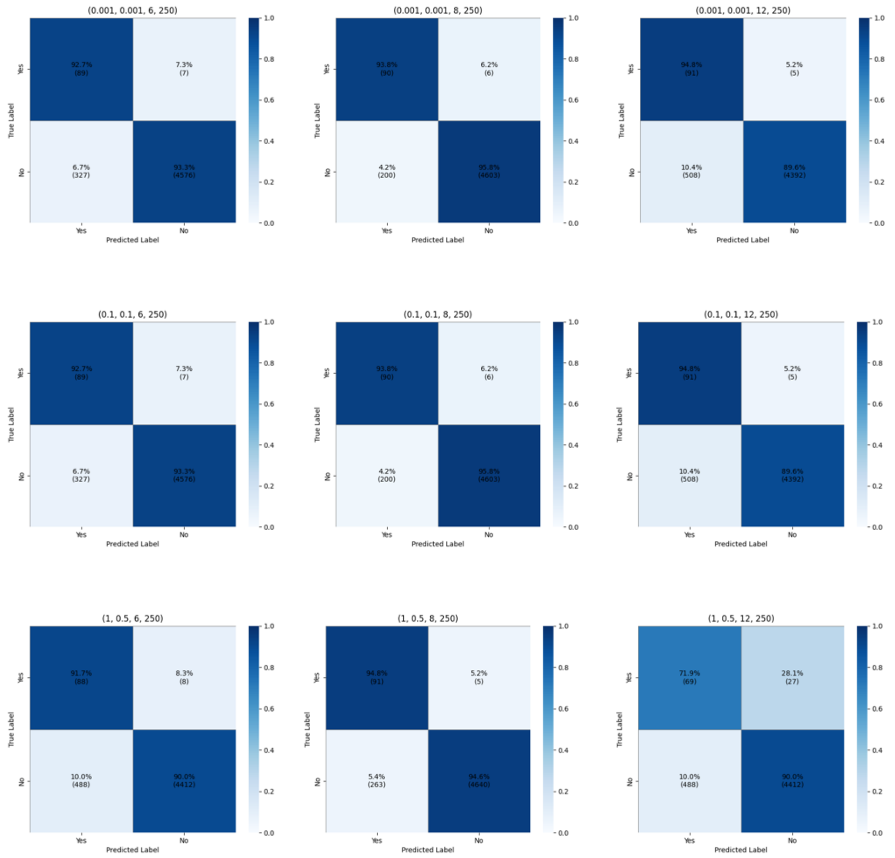

Figure 4.9. Confusion matrix for mortality prediction: with RAG, with 9-shot (MedEmbed).

Confusion Matrix for ASA Classification

Machine Learning method

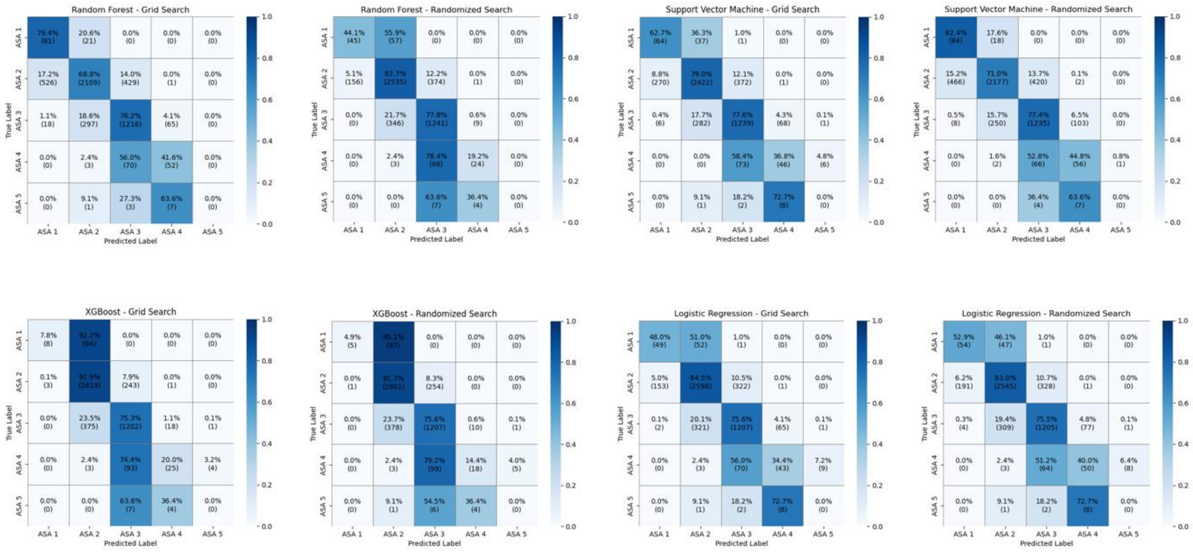

Figure 4.10. Confusion matrix for ASA classification: using machine learning models.

Comparing different LLaMA versions with 5-shot

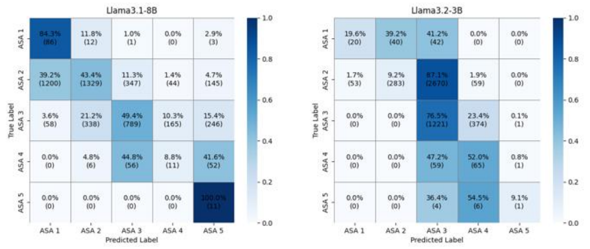

Figure 4.11. Confusion matrix for ASA classification: comparison of different LLaMA versions with 5-shot.

Quantized Llama 3.1-8B

Without RAG and without few-shot (temperature, top_p)

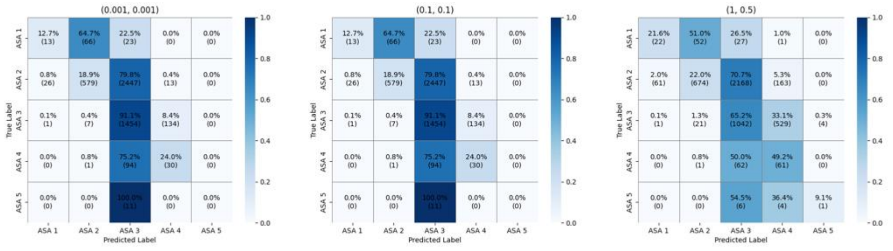

Figure 4.12. Confusion matrix for ASA classification: without RAG, without few-shot.

Without RAG and with few-shot (temperature, top_p)

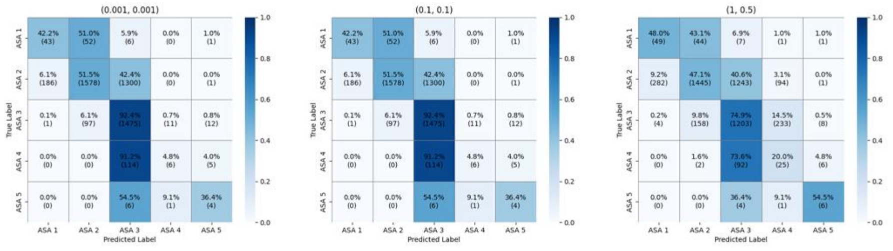

Figure 4.13. Confusion matrix for ASA classification: without RAG, with few-shot.

Confusion Matrix for ASA Classification

Quantized Llama 3.1-8B

With RAG and without few-shot (temperature, top_p, retrieval_top_k, chunk_size) (MedEmbed)

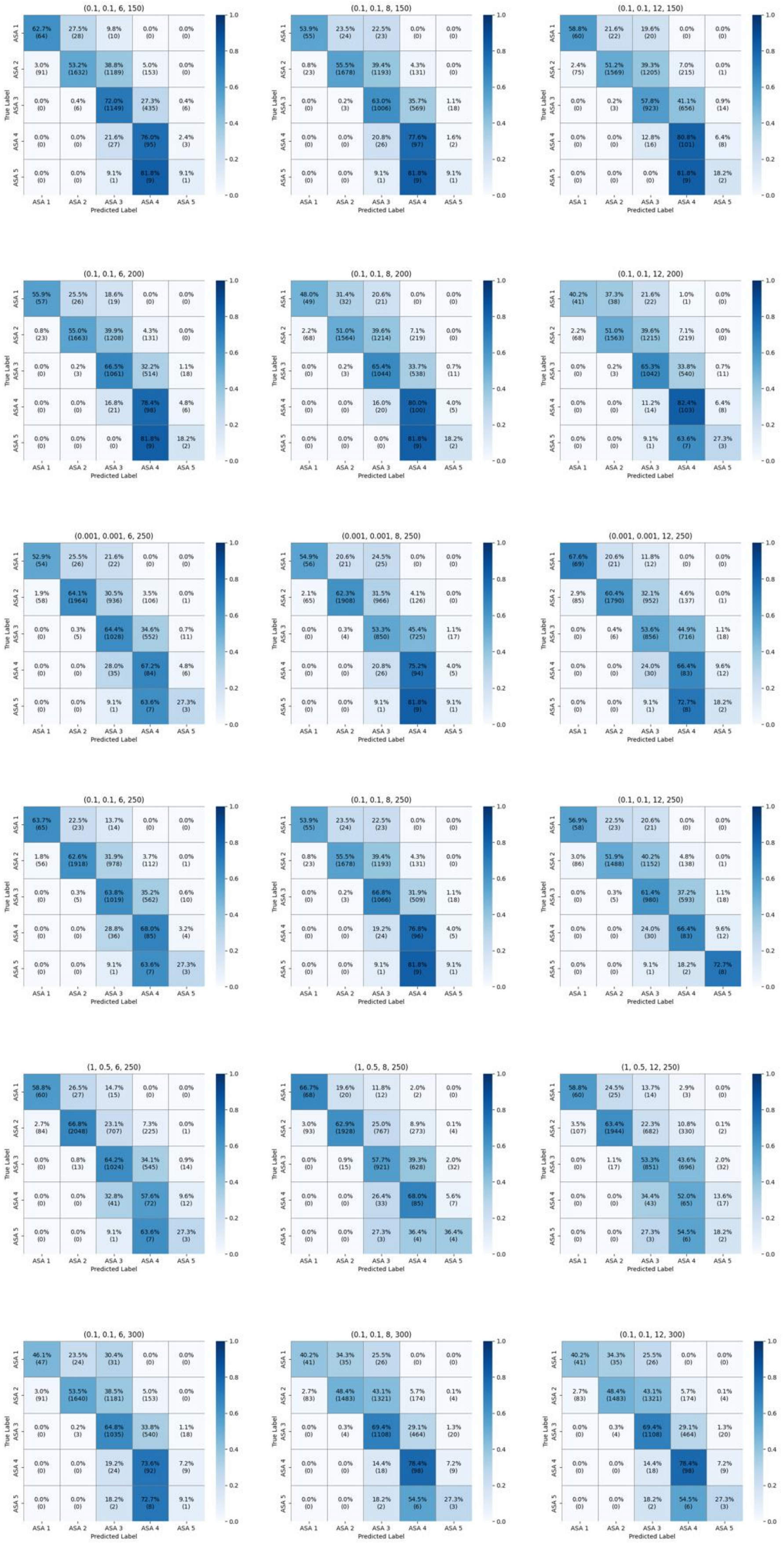

Figure 4.14. Confusion matrix for ASA classification: with RAG, without few-shot (MedEmbed).

Confusion Matrix for ASA Classification

Quantized Llama 3.1-8B

With RAG and without few-shot (temperature, top_p, retrieval_top_k, chunk_size) (MedEmbed)

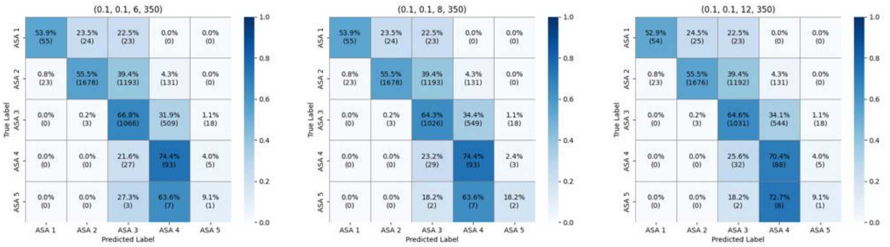

Figure 4.14-2. Confusion matrix for ASA classification: with RAG, without few-shot (MedEmbed).

With RAG and without few-shot (temperature, top_p, retrieval_top_k, chunk_size) (pubmedbert)

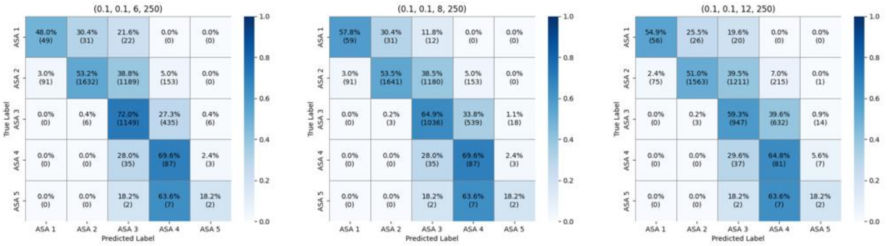

Figure 4.-15. Confusion matrix for ASA classification: with RAG, without few-shot (pubmedbert).

With RAG and with few-shot (temperature, top_p, retrieval_top_k, chuck_size) (MedEmbed)

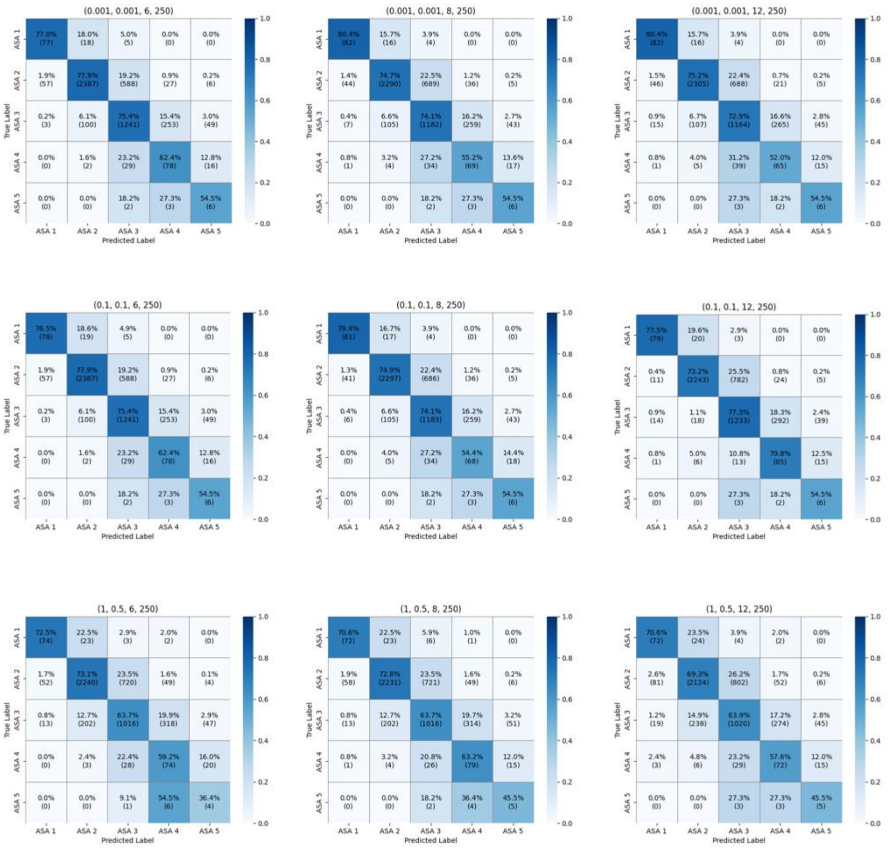

Figure 4.-16. Confusion matrix for ASA classification: with RAG, with few-shot (MedEmbed).
